# Decoding gene regulatory circuitry underlying TNBC chemoresistance reveals biomarkers for therapy response and therapeutic targets

**DOI:** 10.1038/s41698-024-00529-6

**Published:** 2024-03-12

**Authors:** Ryan Lusby, Ziyi Zhang, Arun Mahesh, Vijay K. Tiwari

**Affiliations:** 1 Wellcome-Wolfson Institute for Experimental Medicine, School of Medicine, Dentistry & Biomedical Science, Queens University, Belfast, BT9 7BL UK; 2 Institute of Molecular Medicine, University of Southern Denmark, Odense M, Denmark; 3 Patrick G. Johnston Centre for Cancer Research, Queen’s University, Belfast, BT9 7AE UK; 4 Danish Institute for Advanced Study (DIAS), Odense M, Denmark; 5 Department of Clinical Genetics, Odense University Hospital, Odense C, Denmark

**Keywords:** Cancer, Predictive markers

## Abstract

Triple-negative breast cancer (TNBC) is the most aggressive breast cancer subtype characterised by extensive intratumoral heterogeneity, high rates of metastasis and chemoresistance, leading to poor clinical outcomes. Despite progress, the mechanistic basis of chemotherapy resistance in TNBC patients remains poorly understood. Here, leveraging single-cell transcriptome datasets of matched longitudinal TNBC chemoresponsive and chemoresistant patient cohorts, we unravel distinct cell subpopulations intricately associated with chemoresistance and the signature genes defining these populations. Notably, using genome-wide mapping of the H3K27ac mark, we show that the expression of these chemoresistance genes is driven via a set of TNBC super-enhancers and associated transcription factor networks across TNBC subtypes. Furthermore, genetic screens reveal that a subset of these transcription factors is essential for the survival of TNBC cells, and their loss increases sensitivity to chemotherapeutic agents. Overall, our study has revealed epigenetic and transcription factor networks underlying chemoresistance and suggests novel avenues to stratify and improve the treatment of patients with a high risk of developing resistance.

## Introduction

Triple-negative breast cancer (TNBC) is a highly heterogeneous disease defined by the absence of oestrogen receptor (ER) and progesterone receptor (PR) expression and human epidermal growth factor receptor 2 (HER2) overexpression^1^. It is associated with a poorer clinical outcome due to a lack of early prognostic techniques, high incidences of relapse, metastasis and a lack of targeted therapeutics^2^. In the neoadjuvant setting, chemotherapy is the standard treatment, which includes a combination of taxanes and anthracyclines. However, ~30–50% of patients develop resistance, and their prognosis worsens to 13–15 months survival^3,4^. Despite TNBC being grouped as a single disease, clinical, histological, and molecular profiling have highlighted its intrinsic heterogeneity^5^. This heterogeneity is further highlighted with the identification of unique TNBC subtypes (TNBC type-4 classification) that include: basal-like 1 (BL1), basal-like 2 (BL2), mesenchymal (M) and luminal androgen receptor (LAR)^6^. Each subtype displays unique transcriptional patterns, biology and chemotherapy response^7,8^.

The distal gene regulatory landscape plays a critical role in driving disease-associated altered cell-fates^9^. A super-enhancer (SE) is a cluster of enhancers initially found to be essential in determining cell identity during differentiation but have progressively been implicated in disease initiation and progression, including tumorigenesis^10–12^. In breast cancer, it has been demonstrated that enhancer and SE transcription can reveal insights into subtype-specific gene expression programmes^13^. SEs exhibit high transcription factor density, especially for core regulatory circuitry (CRC) transcription factors (TFs) and drive the expression of key genes that strongly influence cellular identity and function^11,14,15^. These CRC TFs have been shown to self-regulate, where they inwardly bind to SE regions and outwardly regulate SE-associated genes with the CRC, forming a forward-feeding loop. Accordingly, disrupting SE structure or inhibiting SE targeting factors has shown promising results as a potential therapeutic avenue for certain cancers^16,17^. Surprisingly, however, the contribution of SEs and associated CRC landscapes in regulating the gene regulatory programmes underlying TNBC aggressiveness remains unknown. In particular, it remains to be known whether TNBC subtype-specific super-enhancers and CRCs exist to confer different degrees of chemoresistance in these subtypes.

In this study, we aimed to address these longstanding questions by characterising the epigenomic, transcriptomic and TF landscape underlying chemoresistance in TNBC patients. By profiling matched longitudinal single-cell RNA-sequencing data (scRNA-seq) of chemoresponsive and chemoresistant TNBC patients, we identified unique cellular subpopulations associated with chemoresistance and revealed genes that define these subpopulations. Notably, a subset of these signature genes outperformed existing gene panels in classifying pathologic complete response versus persistent residual disease against pre-operative neoadjuvant chemotherapy in TNBC. Furthermore, by analysing data from H3K27ac Chromatin immunoprecipitation followed by sequencing (ChIP-seq) of TNBC subtype patients, we define the SE architecture and CRCs associated with the gene expression programme underlying chemoresistance and reveal several TFs whose depletion can improve the efficacy of chemotherapy across TNBC subtypes.

## Results

### A subpopulation-specific gene expression signature associates with aggressiveness in chemoresistant TNBC patients

**Fig. 1:**
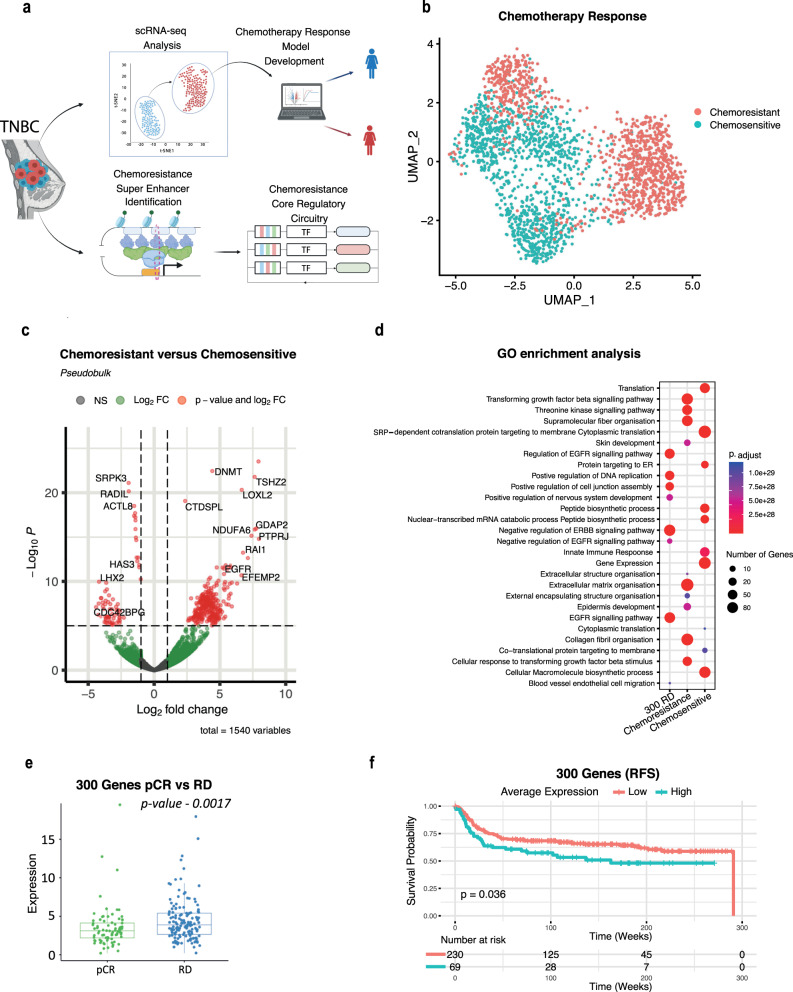
scRNA-seq analysis reveals subpopulations of cells and key genes underlying TNBC chemoresistance. **a** Graphical abstract illustrating the study workflow outlining the comprehensive workflow of our study. Created with BioRender.com **b** UMAP projection showcasing the pre-treatment samples of chemoresponsive and chemoresistant patients. **c** Volcano plot highlighting differentially expressed genes that are significantly upregulated or downregulated in chemoresistant patients, providing critical insights into potential molecular drivers of chemoresistance. **d** Gene Ontology Enrichment Analysis: The Gene Ontology analysis results elucidate the functional significance of markers identified in pre-treatment chemoresistant and chemoresponsive patients. The analysis reveals their substantial involvement in signalling pathways and cell migration, shedding light on the biological processes associated with these genes. **e** Reproducibility analysis demonstrating that the 300 markers identified in pre-treatment chemoresistant patients exhibit higher expression in residual disease compared to pathologic complete response across all bulk RNA-seq datasets (Wilcoxon rank-sum test, *p* = 0.0017). **f** Survival plot depicting the outcomes associated with the 300 genes in TNBC patients from the METABRIC Cohort (Kaplan–Meier, *p* = 0.036).

We began by outlining a stepwise plan to reveal the gene regulatory circuitry underlying chemoresistance in TNBC patients (Fig. 1a). Due to the high degree of intra-tumour heterogeneity associated with TNBC, scRNA-seq provides a higher level of resolution and enables the identification of minor changes in gene expression profiles within tumour cells, being embedded with multiple cell types in a varying proportion which could be lost in bulk RNA-seq analysis. To uncover the underlying mechanisms of chemoresistance in TNBC, we conducted an in-depth analysis of a scRNA-seq dataset initially published by Kim et al.^18^ (SRA: SRP114962). This dataset consisted of 6862 cells sampled from both pre- and post-treatment time points of three patients classified as chemosensitive and four patients classified as chemoresistant to neoadjuvant chemotherapy (NAC) (Supplementary Data 1). It is noteworthy that this dataset exclusively comprised tumour cells, as they were pre-selected using fluorescence-activated cell sorting (FACS) based on aneuploid distributions, before undergoing sequencing (Supplementary Data 1). To classify the tumours as sensitive or resistant in the scRNA analysis, Kim et al.^18^ had performed deep-exome sequencing on 20 patients in which they identified 10 patients where NAC led to clonal extinction (sensitive) and 10 patients where clones persisted (resistant) after treatment. From these 20, Kim et al. selected 7 patients (3 sensitive and 4 resistant) for single-cell RNA sequencing^18^. We hypothesised that by profiling TNBC chemoresistant patient data at the single-cell level, we could identify critical markers driving chemotherapy response. Furthermore, identifying these markers could enable the prediction of chemotherapy response in untreated patients.

As it is thought that chemoresistance occurs due to the clonal evolution of pre-existing clones^18,19^, we focused our analysis on markers unique to the pre-treatment chemoresistant patients to identify the critical transcriptional landscape defining chemotherapy response. In the original study^18^, the authors had highlighted that the batch effects were minimal between patient samples and hence we were convinced we could proceed with merging. In the first instance, using Seurat, we performed SCtransform and scaled each patient’s data before merging to ensure batch effects were minimal across all 7 patients, next we integrated each sample and extracted the cells of the pre-treatment samples and confirmed that batch effects were minimal and gene expression was not affected (Fig. 1b, Supplementary Fig. 1a–d). Clustering analysis of pre-treatment cells revealed that chemoresistant and chemosensitive patients had overlapping clusters, highlighting the lack of batch effects identified by Kim et al., but also a distinct, separate cluster of chemoresistant cells, highlighting a subset of cells that may play a role in patients showing a poor response to chemotherapy (Fig. 1b). Cell annotation analysis, performed by SCSA using established cell type markers from two public databases: CellMarker and CancerSEA. database^20^, revealed that chemoresistant clusters were predominately basal epithelial cells whilst chemosensitive clusters contained luminal progenitor and luminal epithelial cells (Supplementary Fig. 1b). Interestingly, progenitor cells are more likely to be sensitive to anti-cancer therapies^21^, whilst luminal epithelial cells can give rise to basal epithelial cells upon oncogenic stress^22^. Furthermore, whilst the presence of luminal epithelial cells in TNBC tumours may initially appear surprising, it is essential to emphasise that these luminal epithelial cells were indeed tumour cells based on their aneuploid distributions performed by Kim et al. To gain an insight into the transcriptional landscape driving chemoresistance, in pre-treatment patient samples, we applied pseudobulk differential gene expression analysis between chemosensitive and chemoresistance annotations. We identified distinct and statistically significant gene expression patterns for each condition (*p*-value ≤ 0.05 and logfc ≥ 1) (Fig. 1c, Supplementary Fig. 1e). Gene ontology analysis showed enrichment of extracellular matrix remodelling and transforming growth factor-beta (TGF-β) signalling (Fig. 1d), processes associated with EMT, confirming the results from Kim et al. and which have previously been implicated in TNBC chemoresistance^23^. Together these results highlight the existing differential transcriptional landscape of chemoresistant and chemosensitive TNBC patients prior to NAC treatment.

Due to the low patient numbers in the scRNA-seq data, we next sought to identify genes with a reproducible expression in a larger cohort of patients. To address this issue, we obtained and processed bulk RNA-seq datasets (GSE20271, GSE25055, GSE25065, GSE20194 and GSE163882) consisting of 397 TNBC patients, pre-NAC, with known outcomes of pathologic complete response (pCR) and residual disease (RD). To ensure that batch effects between studies were minimal, we corrected using the established R package SVA and the function ComBat which uses empirical Bayes frameworks for adjusting data for batch effects^24^. We found that there were very few batch effects before merging that were corrected to ensure that any residual variations were addressed and did not unduly influence the downstream analysis. (Supplementary Fig. 2a, b). To assess the reproducibility of the genes in a larger patient cohort we compared expression levels of each gene between RD and pCR across 397 TNBC patients total. This resulted in the identification of 300 marker genes which showed a significantly higher expression across all patients with RD (Fig. 1e). By implementing Kaplan–Meier estimator survival analysis on RNA-seq data from the TNBC METABRIC cohort, we revealed that high average expression of these 300 genes is associated with a significantly decreased relapse-free survival in TNBC patients whilst using the median expression as the cut-off point to stratify patients into high and low subgroups (Fig. 1f). In addition, following the reduction in gene numbers due to many having non-detectible expression in bulk RNA-seq data, Gene Ontology analysis revealed that these genes were significantly involved in EGFR signalling pathway (Fig. 1d), which is previously implicated in TNBC chemoresistance^25–27^.

### Distinct transcription factor regulons are active in pre-treatment chemoresistant cells

**Fig. 2:**
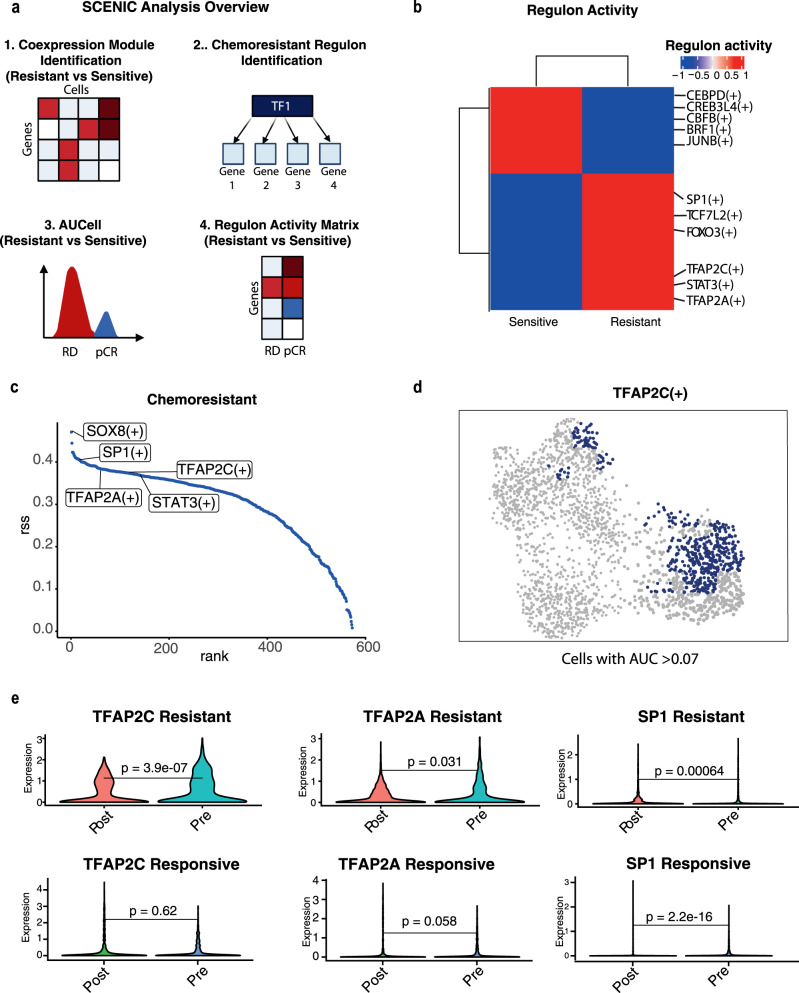
SCENIC analysis reveals potential chemoresistance gene regulons. **a** SCENIC Workflow: (1) Co-expression modules between chemoresistant and chemosensitive cells are identified using GRNBoost. (2) Regulons are then identified using cisTarget. (3–4) The activity of regulons is quantified by assessing the enrichment of the target genes using AUCell. **b** Heatmaps of Significant Top Regulators: Heatmaps display significant top regulators based on the Area Under the Curve (AUC) score for chemosensitive and chemoresistant cohorts. The top motifs, based on averaged binary scores are labelled. **c** RSS Plot of Top Regulons: The regulon set enrichment score (RSS) plot illustrates the top regulons for chemoresistant clusters, with the top motifs prominently highlighted. **d** UMAP visualisation highlighting cells with AUC > 0.07 TFAP2C regulon activity. **e** Violin plots demonstrating the expression levels of TFAP2C, TFAP2A, and SP1 in Resistant and Responsive patients from scRNA-seq data, emphasising higher expression levels that persist following treatment in chemoresistant patients (Wilcoxon rank-sum test).

Currently, the regulatory landscape driving TNBC chemoresistance is unknown. We sought to address this by investigating potential regulatory mechanisms that govern the expression of chemotherapy-resistant genes. By deploying a single-cell regulatory network inference and clustering (SCENIC)^28^ computational pipeline to identify regulons (TFs and their targets) we sought to assess their activity in the chemoresistant cell populations compared to chemosensitive populations (Fig. 2a). In brief, first co-expression modules were identified using GRNBoost. Next, the motifs driving resistant cells were discovered using cisTarget. Finally, the regulon activity was quantified by assessing the enrichment of the regulon target genes using AUCell^29^. Through this analysis, we identified regulons with high activity and specificity scores for both chemoresistant and chemosensitive cells (Fig. 2b, c, Supplementary Data 2). Of note, the Transcription Factor (TF) TFAP2C was identified among the top regulons based on the AUCell score in chemoresistant cells and not present in chemosensitive cells (Fig. 2c, d) and has previously been implicated in EMT signalling and chemoresistance in lung adenocarcinoma, but not yet in TNBC^30,31^. Additionally, we discovered SP1 which was shown to promote chemoresistance and metastasis in ovarian cancer and breast cancer^32^. In both cases, it has been implicated in EGFR transactivation and facilitating migration and invasion through Smad3 and ERK/Sp1 signalling pathways^32,33^. Furthermore, another regulon TFAP2A has also been associated with chemoresistance in colorectal cancer but not yet in TNBC^34^. Interestingly, expression of many of these TFs including TFAP2C, TFAP2A and SP1 were higher in treatment naïve chemoresistance patients as compared to the chemoresponsive patients and these patterns persisted post-chemotherapy (Fig. 2e). To eliminate the possibility of results being driven by a specific patient, we conducted a renewed SCENIC analysis, specifically opting for a per-patient comparison (Supplementary Fig 2c). This reiterated analysis underscored the shared activity of the identified transcription factors (TFs) across all chemoresistant patients, distinctly absent in chemoresponsive individuals. Furthermore, we examined the expression levels of each TF among patients and consistently observed heightened expression in all chemoresistant patients in comparison to their chemosensitive counterparts (Supplementary Fig. 2d). Such higher expression and activity of these TFs in resistant patients compared to sensitive implicates these TFs among the key contributors of chemoresistance in TNBC patients.

### A minimalistic gene signature of 20 genes can predict chemotherapy response in treatment naïve TNBC patients with high accuracy

**Fig. 3:**
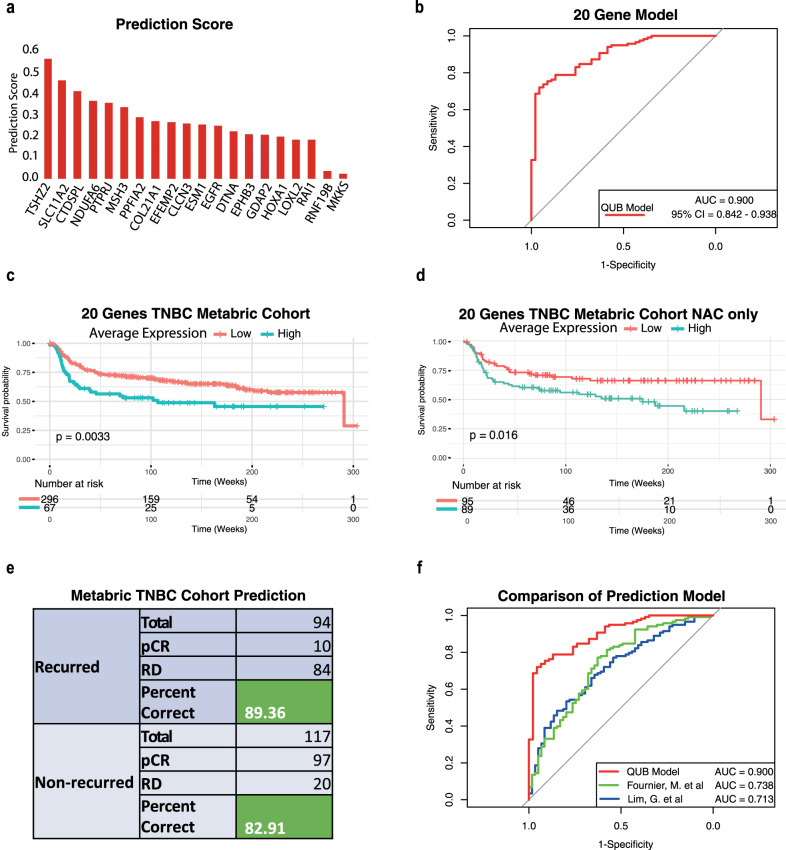
A 20-gene model shows high accuracy in predicting chemotherapy response in TNBC patients. **a** Ranked importance of each gene: Prioritisation of individual gene contributions in predicting response to neoadjuvant chemotherapy (RD) in TNBC patients. **b** ROC curve for model accuracy: receiver operating characteristic (ROC) curve demonstrating the predictive accuracy of our 20-gene model for chemotherapy response in TNBC patients. **c** Survival analysis in METABRIC cohort: Kaplan–Meier survival plot illustrating the survival outcomes of TNBC patients from the METABRIC Cohort based on the expression pattern of the 20 genes (Log-rank test, *p* = 0.0033). **d** Survival analysis in NAC-treated TNBC patients: Kaplan–Meier survival plot revealing the survival outcomes of TNBC patients from the METABRIC cohort who exclusively received neoadjuvant chemotherapy (NAC) based on the expression of the 20 genes (Log-rank test, *p* = 0.016). **e** Prediction of relapse-free survival: predictive assessment of relapse-free survival for each TNBC patient in the METABRIC cohort using our 20-gene model. **f** Model performance comparison: ROC curve depicting the comparative performance of our 20-gene model against previously published prediction models for TNBC chemotherapy response.

In TNBC, patients achieving a pathologic complete response to neoadjuvant chemotherapy is a crucial predictor of a patient’s long-term outcomes and can allow an early evaluation of the effectiveness of systemic therapy^3,35^. We next wanted to investigate whether the genes identified are potentially the critical drivers of chemoresistance in these patients by identifying a significant gene set that could accurately stratify RD and pCR patients. By utilising Lasso and Elastic-Net Regularised Generalised Linear Models, we aimed to identify a significant gene set that could accurately differentiate between pCR and RD in TNBC patients. We derived our training dataset by combining GSE20271 and GSE25055 datasets with 177 TNBC patients (57 pathologic complete response, 120 residual disease), and to derive the validation dataset, we combined GSE25065 and GSE20194 datasets with 130 TNBC patients (46 pathologic complete response, 84 residual disease). We combined these datasets to increase the training and testing cohorts to improve the strength and validity of the proposed gene model as previously shown^36^. In brief, we built a single-fold lasso-penalised model for all genes in the training dataset, then performed 10-fold cross-validation (Supplementary Fig. 3a, b) to identify the best predictors of RD vs pCR. We then took our top predictors (Fig. 3a), built a new model and performed ROC analysis on our validation dataset. This analysis revealed a total of 20 genes (CLCN3, NDUFA6, PTPRJ, GDAP2, RNF19B, MKKS, TSHZ2, COL21A1, LOXL2, SLC11A2, ESM1, CTDSPL, RAI1, EFEMP2, DTNA, EPHB3, EGFR, HOXA1, MSH3 and PPFIA2) to have the strongest discriminatory power between RD and pCR, training AUC = 0.90 (Fig. 3b), Validation AUC = 0.89 (Supplementary Fig. 3c).

As we have been focusing on TNBC only, we next sought to explore the role of the 20-gene model in other breast cancer subtypes. We first investigated the expression of all 20 genes in the TCGA-BRCA dataset containing the four primary subtypes of breast cancers. In combination, the average expression of the 20 genes showed significantly higher expression in the Basal subtype compared to Luminal A and B breast cancer subtypes but lower in the HER2 subtype (Supplementary Fig. 3d). We next wanted to expand the utility of our 20 gene panel to include a prognostic capability through testing 5-year relapse-free survival. Notably, survival analysis of all TNBC patients from the METABRIC cohort, using median expression as the cut-off value, revealed that in combination high expression of this gene signature is associated with significantly reduced relapse-free survival over five years (Fig. 3c). However, in luminal A/B and HER2 patients’, higher expression of the gene set had no correlation with increased or reduced survival (Supplementary Fig. 3e, f). Highlighting that higher expression of these genes in TNBC patients only is underlying their chemoresistance potential. Furthermore, when filtering TNBC METABRIC patients for those only receiving NAC we found that high expression of the 20 genes is again associated with reduced survival irrespective of the chemotherapy regimen (Supplementary Fig 3h). Additionally, we filtered for luminal patients receiving chemotherapy and found again that our gene signature was not predictive in this cohort (Supplementary Fig 3e). Altogether, these findings suggest that increased expression of these genes is specific to TNBC in treatment naïve patients and may drive chemoresistance leading to poor outcomes.

Whilst we had built and tested the model on two large external cohorts we next sought to further validate our model’s predictive strength by applying it to the TNBC METABRIC cohort. Again, whilst not considering which chemotherapy regime was applied and using patients’ relapse-free status as a determination of pCR and RD, our model successfully predicted 89.4% of RD and 82.9% of pCR patients correctly (Fig. 3e). This outcome successfully highlights, not only the predictive strength of our model but also highlights that high expression of the 20 genes can give insights into patients relapse-free survival. Unlike other breast cancer subtypes, there are currently no tests in clinical use for TNBC patients to accurately predict NAC response and facilitate their clinical management^37^. While several predictive panels have been published for TNBC, none have achieved clinical utility due to small sample sizes, lack of validation data and inability to achieve the necessary predictive strength in oestrogen and HER2 positive tumours^7,36,38–40^. Addressing this critical unmet need, we show that our 20 genes hold a high predictive power in determining RD or pCR (with an area under the curve (AUC) of 0.90) (Fig. 3b). Notably, our model utilising this minimal 20 gene set outperformed all previous models in predicting chemotherapy response in TNBC^36,40^ (Fig. 3f). The specific combination of these 20 genes was essential for its high performance as further removal of genes (top 5) significantly reduced the accuracy (AUC: 0.827) (Supplementary Fig. 3g). Additionally, by applying 20 cross-validation iterations we found that the predictive strength remained the same (Supplementary Fig. 3h).

One of the key factors that result in TNBC being the most aggressive breast cancer subtype is tumour heterogeneity. Due to this, recent studies have emerged that have further classified TNBC into four primary subtypes, with each having distinct transcriptional programmes and differing responses to chemotherapy^7,8^. To address this and explore the role and potential of our 20 gene panel in classifying chemotherapy response in scRNA-seq data and within each TNBC subtype we performed pseudobulk RNA-seq analysis, on a dataset containing 6 TNBC patients^41^. We successfully classified each patient into TNBC subtypes; basal-like (BL1 and BL2), luminal androgen receptor (LAR) and mesenchymal (M) using TNBCtype^6^ (Supplementary Fig. 4a, b). To broaden our model’s applicable strength, we applied our prediction model to the pseudobulk data, resulting in the prediction of three patients as having a potential for developing RD (Supplementary Fig. 4c, d). Using the R package “UCell”^42^ we measured the average expression of our 20 genes across each subtype and prediction and found that our signature was higher in patients with BL1 and LAR subtypes and patients predicted to have RD (Supplementary Fig. 4e, f). Furthermore, we employed UCell scoring on the original Kim et al. dataset, revealing that a predominant proportion of cells were identified within the chemoresistant cluster (Supplementary Fig. 4g). This outcome signifies the efficacy of our signature in accurately capturing and discerning cells associated with chemoresistance within the context of scRNA-seq (Supplementary Fig. 4g). Together these findings underscore the precision and success of our selected gene set in recognising molecular patterns linked to chemoresistant phenotypes at the single-cell level. These results suggest that higher expression of a distinct set of genes, originating from specific cellular subpopulations, potentially drives chemoresistance in certain TNBC patients. Overall, our findings revealed a minimalistic gene signature of 20 genes that can predict chemotherapy response in treatment naïve TNBC patients with high accuracy and hold strong potential for prognosis in these patients.

### A distinct epigenetic landscape defines chemoresistance status

**Fig. 4:**
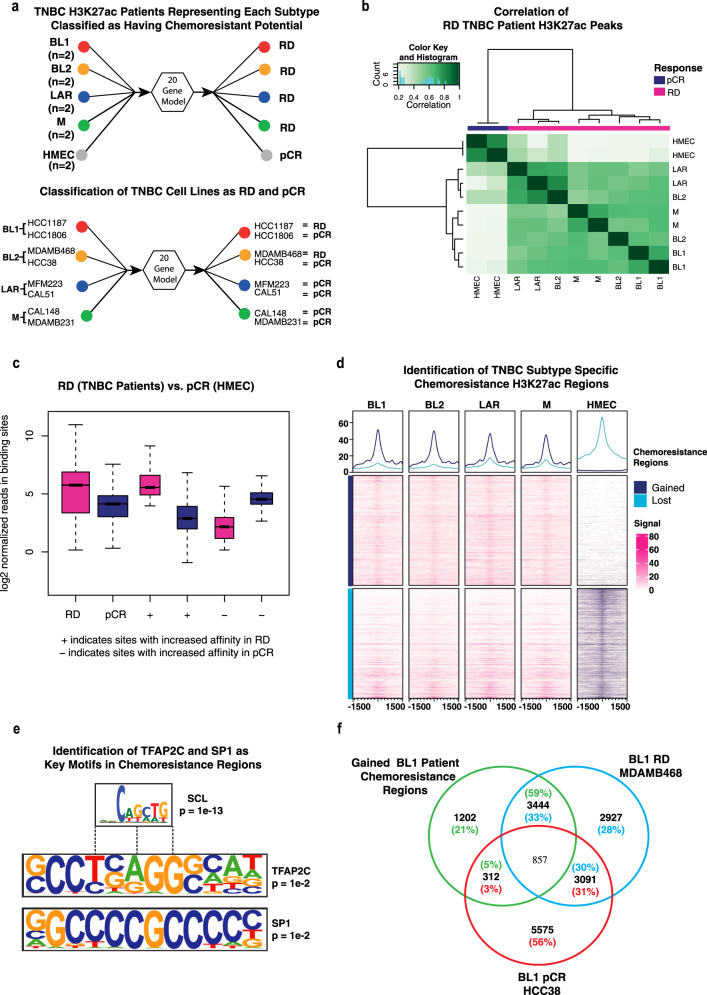
A unique chromatin profile delineates the status of chemoresistance. **a** Chemoresistance prediction: Prediction results illustrating the chemoresistance status of 7 TNBC patients and cell lines representing each TNBC subtype. **b** Correlation of H3K27ac peaks: Correlation analysis of H3K27ac peak profiles between TNBC patients with residual disease (RD) and human mammary epithelial cells (HMEC) classified as pathologic complete response (pCR). **c** Identification of RD-specific H3K27ac peaks: Identification of H3K27ac peaks specific to residual disease (RD) compared to pathologic complete response (pCR). **d** Genomic regions associated with chemoresistant genes: Utilising genomic locations of chemoresistant genes identified in pre-treatment single-cell RNA sequencing data to pinpoint gained regions in each TNBC subtype (RD) that are absent in HMEC (pCR). **e** Key motifs at chemoresistance regions: Identification of TFAP2C and SP1 as key motifs at chemoresistance-associated genomic regions. **f** Comparative Genomic Regions: Comparison of genomic regions gained in chemoresistant BL1 cell line MDAMB468 and lost in chemosensitive BL1 cell line HCC38.

Epigenomic dysregulation is known to play a critical role in disease progression in multiple cancers, including TNBC. The acetylation of Lysine 27 at Histone H3 (H3K27ac) is a mark of active proximal and distal regulatory elements including enhancers and known to govern the gene expression programmes associated with cell identity. Therefore, we analysed ChIP-seq) data for H3K27ac for eight primary TNBC patients as well as corresponding transcriptome (RNA-seq) datasets (ENA: accession number PRJEB33558). First, by applying our therapy resistance prediction model to the RNA-seq data from each patient, we were able to classify each as having a potential for developing RD while normal human mammary epithelial cells (HMEC) as pCR (Fig. 4a). While patient outcomes were unknown for this dataset, we conducted predictions confidently due to extensive validation in the large cohort where the model originated (Fig. 3b and f, Supplementary Fig. 3a–f), and additional validation in the well-documented METABRIC TNBC cohort (Fig. 3e). We next classified each patient sample into four TNBC subtypes, basal-like (BL1 and BL2), luminal androgen receptor (LAR) and mesenchymal (M) using TNBCtype^6^. Using a similar approach, we also classified TNBC cancer cell lines into TNBC subtypes (Fig. 4a). Furthermore, given the tumour-cell exclusive origin of our signature, we also attempted to classify these cell lines as pCR and RD and were successful (Fig. 4a).

We next investigated how the distribution of H3K27ac changes across these subtypes and between pCR and RD. To accomplish this, we assessed the overlap of all H3K27ac peaks within each TNBC subtype, pinpointing regions exhibiting heightened enrichment in RD versus pCR (Fig. 4b, c). Subsequently, for characterising chemoresistance genes using subtype-specific H3K27ac signals, we examined alterations in H3K27ac marks within the regions associated with chemoresistant genes across each subtype and pCR samples. Differential enrichment of peaks between pCR and RD was called using the R package “Diffbind”^43^, with the criteria of a minimum of 50% overlap of peaks. This analysis further showed that there was a significant gain of H3K27ac enrichment at chemoresistance genes across each TNBC sample (RD) and a significant loss in pCR (Fig. 4d). However, whilst there was a noticeable difference in the intensity of H3K27ac activity in these gene regions when compared to HMEC, suggesting that these gene regions are enriched in TNBC subtypes, this analysis was not able to fully discriminate regions that gained sites (with positive fold change) and lost sites (negative fold change) when comparing between TNBC samples. As we had used these criteria to call these peaks (differential enrichment), it is possible that some regions still had sufficient enrichment of H3K27ac that shows up in the heat map even though it is reduced in comparison to the other condition.

To gain insights into the regulatory machinery, we next sought to identify binding sites of specific transcription factors at chemoresistance genes with acquired H3K27ac marks in RD patients. Motif analysis revealed again a strong enrichment for TFAP2C and SP1 motifs among others (Supplementary Data 3), with strongest regulon activity in chemoresistant patients (Fig. 2b–e), clearly implying them as potent drivers of the chemoresistance state (Fig. 4e). Furthermore, chemoresistance genes that gained H3K27ac in BL1 RD patient data showed a stronger overlap with similar genes in the RD cell line compared to the pCR (Fig. 4f), showing a conserved nature of the contribution of these genes and their upstream regulation in chemoresistance across systems.

### Unique super-enhancers are associated with TNBC-subtype-specific transcriptional programmes underlying chemoresistance

**Fig. 5:**
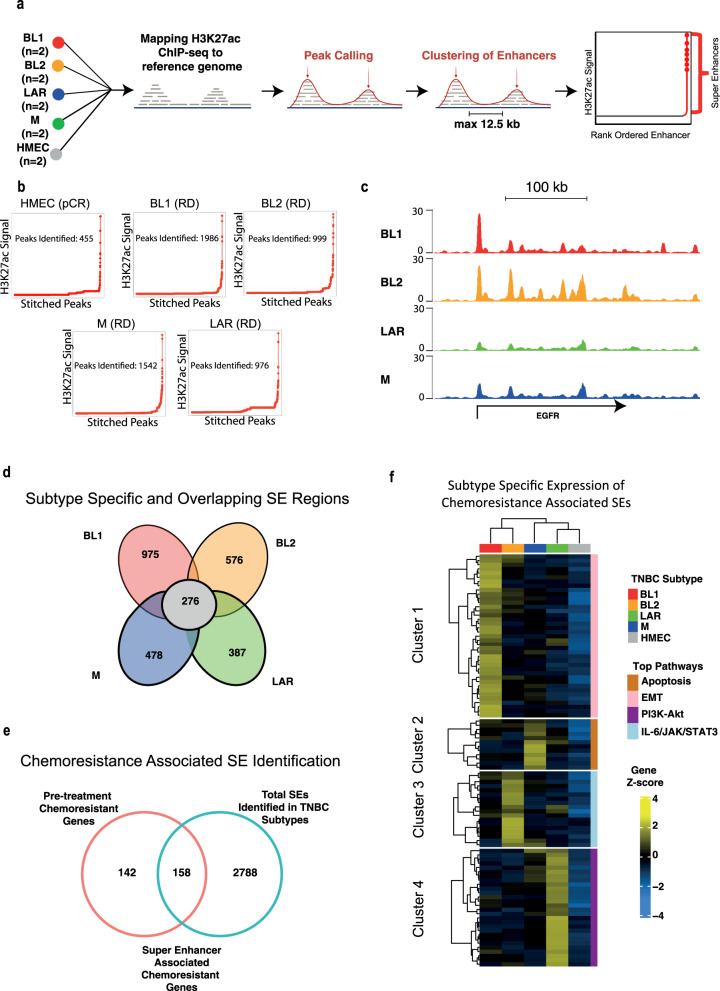
Epigenomic profiling reveals super enhancers in TNBC subtypes which drive the expression of chemoresistance markers. **a** Super enhancer identification workflow: Schematic illustrating the key steps involved in the identification of super-enhancers. **b** Total super-enhancers in TNBC subtypes: ROSE output displaying the cumulative count of super-enhancers identified in each TNBC subtype. **c** Genome browser track of EGFR super-enhancer: Genome browser track highlighting the EGFR super-enhancer identified in BL1 and BL2 TNBC subtypes. **d** Unique and overlapping super enhancers: Visual representation of unique and shared super-enhancer regions across different TNBC subtypes. **e** Super enhancers associated with chemoresistance genes: Identification of super-enhancers (SEs) overlapping with chemoresistance-related genes identified through our reproducibility analysis. **f** SE-associated gene expression heatmap: Heatmap illustrating the unique expression patterns of genes associated with super-enhancers within each TNBC subtype. Unsupervised hierarchical clustering reveals distinct gene expression profiles governed by super-enhancers in each subtype and signalling pathways upregulated by SE-associated genes in each subtype.

Although we demonstrated significant H3K27ac signals for many chemoresistant genes in TNBC patients predicted to have RD, this distinction was not highly discriminative (Fig. 4d). Consequently, our attention shifted towards analysing super-enhancers (SEs), allowing us to characterise subtype-specific features. SEs have increasingly been associated with disease initiation and progression in various contexts, including cancer^15,44,45^. This is particularly interesting as no studies have yet investigated their contribution to TNBC chemoresistance. We, therefore, subjected our genome-wide H3K27ac profiles for TNBC patients to the identification of SE elements. SEs were mapped and quantified by Rank Ordering of Super-Enhancers (ROSE) software. In summary, ROSE analysis was performed with default parameters of 12.5 kb stitching distance, and TSS exclusion size set to 0, with the genome set to hg38^46^. SE-associated genes were identified as the “nearest gene” output from ROSE. Samples were merged based on their subtyping, to identify common subtype-specific SE regions (Fig. 5a), resulting in an average of 1279 SEs identified per tumour sample (Fig. 5b). The genome-wide distribution of H3K27ac SE peaks showed its distribution mostly at intron (59%) and intergenic (33%) locations (Supplementary Fig. 5a).

In the first instance TNBC SE regions were compared with HMEC pCR samples to identify TNBC-specific SE regions (Supplementary Fig. 5b). Further analysis of these data identified 2692 unique and 276 overlapping SEs between each TNBC subtype (Fig. 5c, d). We hypothesised that these subtype-specific SEs govern the expression of a selected set of our marker resistance genes to drive TNBC chemoresistance. Interestingly, of our 300 marker genes, 158 were in close proximity to the discovered subtype-specific SEs (Fig. 5e). Next, we calculated the correlation of expression of SE-associated genes across all patients and performed unsupervised hierarchical clustering to identify SE-associated genes that show subtype-specific expression (Fig. 5f). This analysis identified four prominent clusters with unique characteristics of each subtype, and which showed no expression in HMEC cells. Interestingly, further Gene Ontology analysis showed enrichment of specific pathways in each of these subtype-specific clusters. For example, cluster 1, consisting of BL1-specific SEs, was enriched with EMT-related signatures; cluster 2, consisting of M-specific SEs, was associated with Apoptosis related signatures; cluster 3, consisting of BL2-specific SEs, was enriched for IL-6/JAK/STAT3 signalling signatures while cluster 4, consisting of LAR specific SEs, showed PI3K-Akt related signatures (Fig. 5f, Supplementary Fig. 5c). Of note, EGFR and RAI1, two markers we identified to have a high discriminatory effect in chemoresistant patients, were located in close proximity to a distinct set of discovered SEs in BL1 and BL2 subtypes. Furthermore, our analysis showed that the subtypes LAR and BL1 exhibit the highest number of chemoresistant SEs (Fig. 5f). These findings are in line with previous research, where LAR followed by the BL1 subtype showed the worst response to chemotherapy^47^. To confirm that these SE regions were communicating with the predicted target chemoresistance genes we processed existing Hi-C datasets from TNBC patients^48^. By searching the SE regions output by ROSE, we indeed confirmed that many SEs of interest are looping in close physical proximity to their predicted target genes, including EGFR and RAI1 (Supplementary Fig. 5d). Altogether, these observations suggest that the super-enhancer landscape plays a key role in the evolution of chemoresistance by governing the expression of key driver genes/pathways in a TNBC subtype-specific manner.

### Distinct transcription factor core regulatory circuitries operate at TNBC subtype-specific super-enhancers associated with chemoresistance

**Fig. 6:**
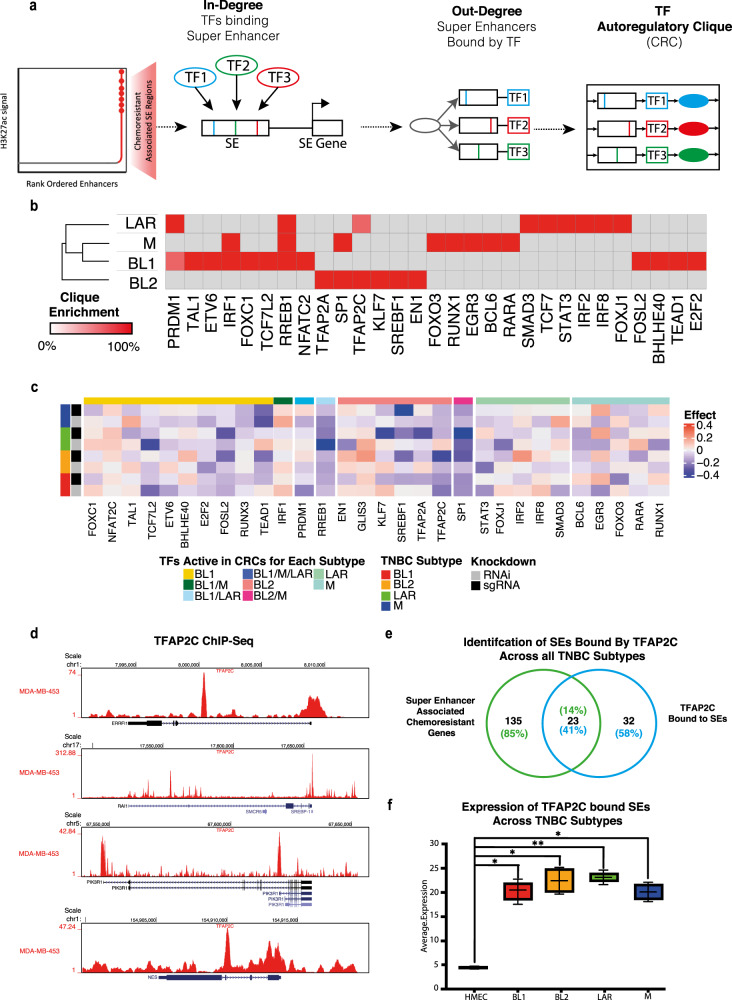
SE–TF connectivity analysis defines core regulatory circuitry underlying TNBC chemoresistance. **a** SE-based CRC analysis: Schematic outlining the SE-based core regulatory circuitry (CRC) analysis. For each TF linked to a chemoresistant SE, in-degree values are calculated through motif identification, while out-degree values are determined for each TF connected to a chemoresistant SE by assessing all other bound SEs at each TF gene locus. Node connections among TFs are employed to identify auto-regulatory cliques that govern the chemoresistant SE network. **b** Heatmap of Clique Enrichment Scores: Heatmap displaying clique enrichment scores for the union of all TFs associated with top SEs across all TNBC samples. Grey boxes represent instances where a TF is not associated with a particular TNBC patient sample. TFs and samples are clustered using Euclidean distance. **c** Subtype-specific genetic dependencies: Heatmaps illustrating the subtype-specific genetic dependencies of each CRC TF, as determined through whole-genome RNA interference (RNAi) and CRISPR screens from the Broad DepMap. Significant subtype-specific genetic dependencies are highlighted, employing a modified *T*-test corrected for multiple hypothesis testing (*T*-value, FDR < 0.1). Blue corresponds to the greater effect of KD influencing genetic dependency. **d** TFAP2C Occupancy Visualisation: UCSC Genome Browser visualisation of TFAP2C occupancy using ChIP-seq data at SEs predicted to be regulated by TFAP2C. **e** Identification of TFAP2C-Bound SEs: Identification of SEs bound by TFAP2C across various TNBC subtypes. **f** Expression of TFAP2C-bound SEs: Expression profiles of SEs bound by TFAP2C across each TNBC subtype and human mammary epithelial cells (HMEC).

Super Enhancers recruit a high density of cell type-specific master TFs to drive cell-state-specific gene expression profiles^49^. Furthermore, the expression of TFs that bind SEs is often regulated by the activity of SEs in a forward feedback loop and is well-established in many malignant cell types^32,33^. To reveal critical master TF interactions responsible for driving the TNBC subtype-specific transcriptional programme associated with chemoresistance, we modelled transcriptional regulatory networks mediated by SEs utilising the Python package “CRCmapper”. It scans TF motifs inside chemoresistance SE regions and then identifies both TFs binding within SE regions and outward binding of SE-associated genes in a complete regulatory circuitry (core regulatory circuitry (CRC) cliques)^50–53^. CRC cliques are then scored based on TFs which exhibit a high frequency of occurrence across each CRC clique, and the top CRC for each TNBC subtype is designated (Fig. 6a).

Following “CRCmapper” analysis on each sample, we calculated a clique enrichment score (the percentage of each CRC in which a TF is a constituent member) (Fig. 6a). Following the scoring of each CRC, we clustered samples based on their clique enrichment scores and revealed intrinsic CRC differences in top TFs across TNBC subtypes and additionally highlighted overlapping TFs common between all subtypes (Fig. 6b, Supplementary Fig. 6a, b). The TFs identified in each TNBC CRC clique include known lineage-defining TFs such as EN1^54^. Other TFs, with a high clique score, include EGR3, STAT3, ETVS, IRF1, IRF2, and IRF8. Additionally, we performed CRC analysis using HMEC SE data to identify the top CRC in normal (pCR) (Supplementary Fig. 6c). Of note, FOXC1 was also discovered among other TFs to be a CRC TF in BL1 in line with the recent findings of it being a SE master regulator of invasion, metastasis and chemoresistance in TNBC^55,56^.

We next sought to explore the role of identified TNBC-subtype CRC TFs in exhibiting strong genetic dependency across multiple TNBC subtypes Our goal was to pinpoint potential candidate TFs with implications for driving chemoresistance across subtypes, holding promise for novel therapeutic interventions. Utilising viability data obtained from the Broad DepMap project, which involved RNAi and CRISPR knockdown experiments, we collected data for each CRC TF across cell lines representing various TNBC subtypes. Subsequently, we conducted a regression analysis to examine the correlation between cell line viability and each subtype, following the loss-of-function assays carried out within the Broad DepMap cohort. This analysis revealed CRC TFs which negatively and positively affected the viability of each TNBC cell line and identified key genetic dependencies specific to each subtype and across all, enabling prioritisation of TFs shown to have a strong genetic dependency across all TNBC subtypes (Fig. 6c). Of note, TFAP2C and SP1 were discovered to be essential for viability across all TNBC subtypes. Other TFs, such as STAT3, show genetic dependency across all subtypes, however in some subtypes, they are stronger when compared to others possibly due to their engagement in other networks (Fig. 6c). Furthermore, RREB1 has been shown to be a critical integrator of TGFβ and Ras signalling pathways during both developmental and cancer EMT programmes^57^.

Based on our prior findings, which included a significant enrichment of TFAP2C motifs at super-enhancers associated with chemoresistance genes (Supplementary Data 3), its elevated regulon activity, and increased expression in chemoresistant patients (Fig. 2b–e), as well as its notable genetic dependency in TNBC cells, we were compelled to delve deeper into the role of TFAP2C in driving TNBC chemoresistance. Leveraging the Super-Enhancer Archive^58^ we initiated the identification of super-enhancers (SEs) and their respective transcription factors (TFs). We subsequently overlaid these findings with our CRC and ROSE analysis results, leading to the generation of a tentative list of top CRC TFs predicted to be bound by TFAP2C. To identify direct targets of TFAP2C, we processed TFAP2C ChIP-seq from the TNBC cell line MDA-MB-453^59^. A visualisation of TFAP2C binding at predicted SE regions indeed confirmed its strong enrichment at these locations (Fig. 6d). Importantly, the SE RAI1, a top chemoresistance signature gene in our prediction model, showed a significant occupancy by TFAPC. We next overlapped all SEs bound by TFAP2C across TNBC patients (*n* = 55) with all SE regions associated with chemoresistance genes, resulting in a total of 23 chemoresistance SEs occupied by TFAP2C (Fig. 6e). Notably, these loci included our chemoresistance signature genes as well as other potentially interesting candidates (examples shown in Fig. 6d). Interestingly, gene expression analysis of chemoresistance genes associated with TFAP2C bound super-enhancers showed that they were expressed at significantly higher levels in all TNBC subtypes as compared to the healthy control cells (HMEC) (Fig. 6f). Altogether these observations highlight that distinct transcription factor CRCs operate at TNBC subtype-specific super-enhancers associated with chemoresistance genes and TFAP2C holds potential as one of the key TFs of this process across all TNBC subtypes.

### TNBC-type specific CRC TFs are essential for the viability of TNBC cells, and their loss enhances sensitivity to chemotherapy

**Fig. 7:**
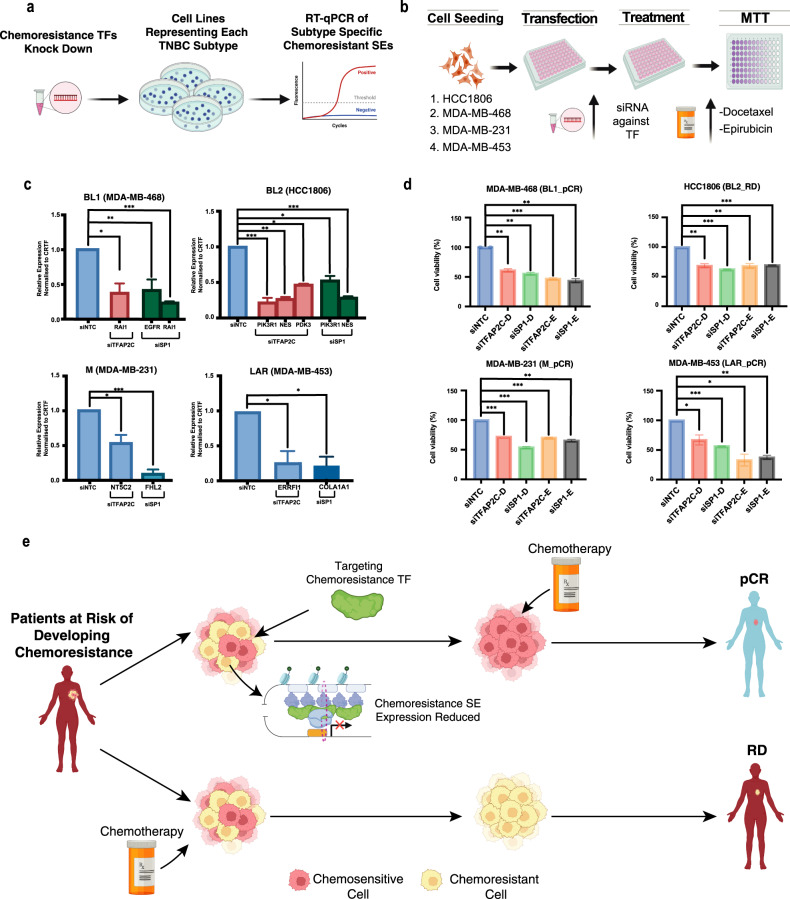
TNBC-type specific CRC TFs are essential for TNBC cell survival, and their depletion improves chemotherapy response. **a** Depletion of selected TFs: Schematic representation of the depletion of specific TFs in cell culture, followed by RT-qPCR experiments to assess the impact on SE-associated gene expression. Created with BioRender.com. **b** RT-qPCR analysis: RT-qPCR analysis of subtype-specific chemoresistant super-enhancers (SEs) following knockdown (KD) of TFAP2C and SP1. Statistical significance was determined using a Student’s *t*-test. Error bars are defined as standard deviation. **c** Workflow for TF depletion: an overview of the workflow for depleting CRC TFs in selected cancer cell lines Created with BioRender.com. **d** Improved cell viability: Reduced cell viability observed following chemotherapy treatment in TNBC subtype-specific cell lines when combined with TF depletion. Statistical significance was determined using a Student’s *t*-test. **e** Enhanced chemotherapy efficacy: Targeting TFs associated with chemoresistant SEs has the potential to eliminate subpopulations linked to chemoresistance, thereby improving the efficacy of chemotherapy. Created with BioRender.com.

We next sought to experimentally investigate whether the predicted CRC TFs actively control the expression of chemoresistance genes and consequently chemotherapy response. Our results revealed that TFAP2C is potentially a master regulator across all TNBC subtypes in driving chemoresistance genes by targeting their SEs. Furthermore, equally interesting was SP1 which similarly also showed a strong enrichment at SEs of chemoresistance genes and high regulon activity and expression in chemoresistance cells. We, therefore, performed depletion of TFAP2C and SP1 in four TNBC cell lines representing each TNBC subtype and measured expression of target SE-associated genes using RT-qPCR assays (Fig. 7a and Supplementary Fig. 6d). Interestingly, in all types, knockdown of TFAP2C and SP1 led to a significant decrease in the expression of genes associated with their target chemoresistance SEs (Fig. 7b). Furthermore, we propose a conceptual model wherein chemoresistant patients exhibit elevated expression of chemoresistance-related genes, such as EGFR and RAI1. Exclusive activation of Chemoresistance SEs is observed solely in chemoresistant patients, with no activity in sensitive counterparts. Specific TFs, including SP1, TFAP2C and TFAP2A are uniquely expressed and selectively bind to these SEs in chemoresistant patients. The orchestrated interplay between the active SE and TFs increases the expression of pivotal chemoresistance genes such as EGFR and RAI1 within this patient subgroup (Supplementary Fig. 6e). Through SCENIC analysis we show that these genes are intricately connected in a network, where each TF binds at SEs of genes shared with our 20 gene model, collectively contributing to the aggressive phenotype associated with chemoresistance (Supplementary Fig. 6e). However, additional studies are necessary to delve into the underlying mechanism of this upregulation.

Given our discovery that numerous SEs are bound by TFAP2C and SP1 and exhibit substantial genetic dependency across all TNBC subtypes, we proceeded to assess whether the downregulation of these TFs would enhance the response to chemotherapy by diminishing the expression of chemoresistance genes. To investigate this, we conducted siRNA knockdown experiments targeting each candidate TF in four cell lines, each representing a distinct TNBC subtype. These experiments were coupled with treatment using two chemotherapy agents, Docetaxel and Epirubicin, and cell viability was quantified through MTT assays (Fig. 7c). Since these TFs may have other physiological roles^34,54,60–63^ in TNBC beyond the context of chemotherapy, we did not characterise the effect of depleting these TFs alone, without the drugs. Notably, the depletion of TFAP2C and SP1 resulted in a marked decrease in cell viability after chemotherapy treatment in all TNBC subtype cell lines compared to the control cells (Fig. 7d). Particularly, TFAP2C knockdown exhibited a significant reduction in cell viability across all subtypes, underscoring TFAP2C as a pivotal and adaptable regulator of chemoresistance throughout all TNBC subtypes. In summary, our findings suggest that a distinct group of TFs likely steer the epigenomic landscape and dictate the gene expression patterns characterising chemoresistance-related cell subpopulations. Moreover, the targeted inhibition of these chemoresistance-associated TFs presents a promising avenue to enhance patient outcomes across all TNBC subtypes (Fig. 7e).

## Discussion

Neoadjuvant chemotherapy (NAC) is used frequently in the treatment of TNBC patients due to the lack of targeted therapeutics and its ability to reduce tumour size, improve surgical outcomes and increase survival in responders. However, due to the intratumoral heterogeneity (ITH) associated with TNBC, patients have differing responses to NAC^64^. Achieving pCR is associated with significantly improved survival outcomes in TNBC patients^65^. Identifying those patients who will have RD following NAC will enable physicians to determine the best therapeutic option at the beginning of treatment, rather than waiting for NAC treatment results, to increase the chances of achieving pCR. Numerous efforts have been put into developing predictive signatures in TNBC, but currently, there is no clinically recommended predictive biomarker panel for NAC response^8,66,67^. However, these studies have focused on bulk RNA-based techniques, in small patient cohorts to identify markers to predict therapy response and do not account for the ITH associated with TNBC.

Here, by profiling chemoresponsive and chemoresistant patients at the single-cell level to identify markers associated with chemotherapy, we have developed a predictive model that has high accuracy in defining chemotherapy response in TNBC patients. Our utilisation of scRNA-seq was not aimed at an exhaustive analysis of chemoresistance but rather focused on defining markers. Notably, Kim et al. had conducted a comprehensive analysis in their study. Our emphasis was on identifying markers specific to pre-treatment chemoresistant cells, utilising single-cell resolution to pinpoint molecular characteristics associated with this phenotype. This approach provides valuable insights into understanding chemoresistance in TNBC without undertaking an exhaustive analysis of individual cell responses, building upon the foundational work by Kim et al. Our 20-gene model, through the identification of markers in scRNA-seq and validation in over 300 patients, holds a high potential for aiding in the clinical management of TNBC patients by enabling the assessment of NAC response upfront. Additionally, we have demonstrated that it outperforms all existing signatures for predicting chemotherapy response in TNBC. Furthermore, higher expression of our models’ genes is also associated with reduced survival and could accurately predict the chemoresistance potential in TNBC patients from the METABRIC cohort. It is strongly associated with EGFR signalling, which has been shown to play a critical role in TNBC chemoresistance^25–27^.

The predictive strength of our model’s combination of genes was further demonstrated by predicting chemotherapy response in the eight untreated TNBC patients with H3K27ac data. Whilst patient outcomes were not known for these samples, our past analysis in model development (Fig. 3b and Supplementary Fig. 3e) and validation in the METABRIC TNBC cohort (Fig. 3e), in cohorts where patient outcomes were well-documented, gave us great confidence in the accuracy of predicting each sample as RD and HMEC and pCR. This provided the unique opportunity and the rationale to map and quantify enhancers genome-wide to shed light on the previously uncharacterised SE landscape underlying chemoresistance in a subtype dependant manner for the first time. By overlapping with chemoresistance genes identified to have a reproducible expression in bulk RNA data, we could identify a subset of chemoresistance genes in close proximity to SEs. Interestingly, BL1 and LAR subtypes had the highest proportion of SEs. LAR followed by BL1 are the top two TNBC subtypes associated with increased chemoresistance and poorer outcomes^47^. The SE-associated genes were significantly involved in EMT and PI3K-Akt signalling processes in BL1 and LAR subtypes. Both have previously been implicated in chemoresistance in multiple cancer types, including breast cancer and specifically TNBC^68,69^.

Additionally, whilst the dysregulation of gene expression in TNBC has been previously characterised, it has not been adequately explained, in which previous studies have not fully elucidated or comprehensively uncovered the underlying gene regulatory circuitry including key transcription factors responsible for the observed dysregulation of gene expression programmes in TNBC by standard or conventional transcriptomic analysis, such as differential gene expression analysis alone. Instead, such extensive changes are attributed to the widespread transcriptional rewiring occurring in breast cancer cells, including the utilisation of core transcription factors, as well as the activation of many gene-regulatory elements, including enhancers and super enhancers^13,70^. Defining epigenomic characteristics is instrumental to dissecting gene regulatory programmes which underlie cancer disease progression. Here, for the first time, we have profiled and characterised the TF regulatory network, using subtype-specific SE profiles, underlying TNBC chemoresistance. This systematic identification of active TNBC subtype regulatory elements has led to several enabling observations. By constructing the TNBC TF regulatory network using subtype-specific SE profiles, we can identify the critical TF nodes that enforce the TNBC subtype epigenome underlying chemoresistance. Of note, TFAP2C, TFAF2A, and SP1 were shown to have higher expression in chemoresistance pre- and post-single-cell data, highlighting their implication in driving chemoresistance in TNBC. Additionally, by profiling TFAP2C ChIP-seq in a TNBC cell line we found that many chemoresistance-associated SEs, including RAI1, were bound by TFAP2C, establishing its direct function in driving TNBC chemoresistance. Furthermore, we depleted key chemoresistance TFs predicted to function at subtype-specific chemoresistance SEs of chemoresistance genes and measured their expression using RT-qPCRs (Fig. 7b). These results show a clear, significant reduction in the expression of target chemoresistance genes, validating our proposal for the role of these TFs in regulating their expression. These results are also in line with our observations that the depletion of these TFs can significantly overcome chemoresistance (Fig. 7d). Altogether, these observations conclude that chemoresistance is governed by a distinct set of genes that are controlled by CRC TF networks through a subtype-specific set of SEs. Additionally, they were shown to have a high genetic dependency in each TNBC subtype cell line. Of note, suggesting that inhibition may provide an approach to overcome chemotherapy resistance in all TNBC subtype tumours. In recent years, targeting TFs using small molecules that bind to specific nuclear hormone receptors has proven to be successful in many cancers^71^, in particular, the SP1 inhibitor Mithramycin A has been shown to inhibit and suppress cell survival in in vitro models of basal TNBC^72^. Along these lines, targeting TFAP2C may dramatically improve chemotherapy efficacy in patients with a high risk of chemoresistance.

In BL1, FOXC1 was highlighted as one of the top TFs driving chemoresistance SEs. In the single-cell data, FOXC1 was shown to have higher expression in chemoresistant cells pre- and post-chemotherapy. FOXC1 has recently been shown to be a master TF, encoded by SEs in TNBC^55^. Additionally, TFAP2C has never been shown to drive SE expression in TNBC, nor has it previously been implicated in TNBC chemoresistance. Its key role in potentially driving TNBC chemoresistance is further highlighted by our SCENIC analysis. We identified TFAP2C, along with several other CRC TFs, as key regulons in defining the chemoresistance subpopulations in the scRNA-seq data. While it was identified as a core TF in BL2, we have demonstrated it has a high genetic dependency and potential regulator of chemoresistance SEs in all TNBC subtypes. Additionally, it has been implicated in chemoresistance in several cancers^29,73^ and, notably, Docetaxel resistance in lung adenocarcinoma^74^. The TFs TFAP2C and SP1 were identified throughout our study from the single-cell to CRC analysis as potentially having a significant role in driving chemotherapy resistance-associated gene expression programme. We have successfully shown that direct targeting of these TFs has the potential to increase the efficacy of chemotherapy agents across each TNBC subtype. Whilst there are no clear TFs that act in a unique subtype dependant manner, we have shown that the TF TFAP2C is a master regulator of subtype-specific chemoresistance SEs across all TNBC subtypes resulting in potential for the development of novel therapeutics that can aid in improving the efficacy of NAC.

Our results have clearly highlighted how a better understanding of gene regulatory circuitry allows identifying novel therapeutic avenues. This study creates the rationale for further functional studies to determine their mechanistic roles in chemoresistance and potentially lead to the development of novel targeted therapeutics. Additionally, as the model was developed based on a combination of NAC, it may be possible to extend its application range to develop drug-specific or secondary therapeutic prediction models and further stratify TNBC patients. One potential limitation of our study is the low number of patient samples for SE identification. However, the genes identified in close proximity to SEs were shown to have higher expression in RD TNBC patients across multiple studies and TNBC subtype-specific cell lines, validating their role in TNBC chemoresistance.

In summary, we reveal cell subpopulations associated with TNBC chemoresistance and the signature genes defining these populations of which a subset acts as a best-in-class gene signature for an accurate prediction of chemotherapy response. Notably, we show that these chemoresistance genes are controlled by a specific set of transcription factor networks and super-enhancers in a TNBC-subtype-specific manner. Importantly, we demonstrate that targeting these TFs holds the potential to overcome chemoresistance and ultimately improve patient survival.

## Methods

### Identification of chemoresistant cell types using single-cell RNA-sequencing analysis

To identify cell types and their markers associated with TNBC chemoresistance, scRNA-seq analysis was performed on the data set obtained from Kim et al.^18^, consisting of matched pre and post-chemotherapy (anthracycline and a taxane) samples from four responsive and four resistant patients with a total of 6,862 cells. Raw scRNA-seq data underwent quality control, including library size, mitochondrial gene content, and UMI detection. Cells failing quality control criteria were excluded. The ‘sctransform’ method from Seurat v3 was applied for normalisation to address technical variability across samples. The integrated dataset was generated using the ‘FindIntegrationAnchors’ and ‘IntegrateData’ functions from Seurat v3. Subsequently, the data were scaled using the “ScaleData” function from Seurat v3. The analysis pipeline post-integration included variable feature selection, principal component analysis (PCA) for dimensionality reduction, and clustering using the ‘FindClusters’ function in ‘Seurat’. Uniform manifold approximation and projection (UMAP) was employed for the visualisation of clustered cells. Cluster annotation was performed using the Python programme “SCSA” to identify associated cell types and cancer-related processes. All significantly expressed markers for each treatment time point and therapy response were polled to identify uniquely expressed markers in pre-chemoresistant patients that could potentially have a crucial role in driving TNBC chemoresistance.

### Reproducible signature marker identification

For the reproducibility of the gene set identified using scRNA-seq analysis, we used five independent bulk RNA-seq datasets, GSE20271, GSE25055, GSE25065, GSE20194, GSE163882, of 397 TNBC patients where their chemotherapy response was available (RD, PCR). Patient samples were excluded if the therapeutic outcome (residual disease or pathologic complete response) was unknown and not classified as TNBC. The raw data was normalised, batch corrected, and log-transformed using the R package “affy” and “TDM and the Python package “pyComBat”. In total, 397 patient’s data were selected for reproducibility analysis. The 300 genes identified in the scRNA-seq dataset were extracted from the normalised bulk RNA-seq count files. A custom R script was used to compare each gene’s expression in patients with residual disease and pathologic complete response, Wilcoxon Rank Sum and Kruskal-Wallis tests were used to calculate significance.

### Pseudobulk analysis

All six patient data files were downloaded from: GSE118390 and the chemoresistant scRNA-seq was analysed using the same parameters in the R package “Seurat”. First, cells with feature counts of greater than 2500 or less than 200 were removed, including mitochondrial reads of greater than 5%. Following the removal of cells, downstream analysis, including normalisation, variable feature selection, dimensionality reduction and UMAP clustering, was performed. Signature scoring was performed by the R package UCell^42^ using default parameters. Following downstream analysis, pseudobulk analysis was performed using the R package SingleCellExperiment and the function “AggregateExpression”.

### Implementation of GENIE3 and SCENIC

Single-Cell regulatory Network Inference and Clustering (SCENIC) analysis was performed to reveal the core TFs in chemoresistant and chemosensitive clusters^28^. We performed the SCENIC analysis using the latest version of pySCENIC. The gene-motif rankings (500 bp upstream or 100 bp downstream of the transcription start site) were used to determine the search space around the TSS. The motif database was used for RcisTarget and GENIE3 algorithms to infer the core TFs. Wilcoxon rank sum and Kruskal–Wallis tests were used to calculate significance.

### Identification of significant gene set and construction of the prognostic prediction model based on residual disease vs. pathologic complete response

Raw microarray expression (CEL) files of all 310 TNBC patients were downloaded from Gene Expression Omnibus, GSE20271, GSE25055, GSE25065 and GSE20194. Gene expression profiles were quantile normalised and log2-transformalised using “BART”, followed by batch correction using “ComBat” from the R package “sva”. To identify the most significant gene set, GSE20271 and GSE25055 datasets with 177 TNBC patients (57 pathologic complete response, 120 residual disease) were used to build the model. To verify the strength of the geneset, GSE25065 and GSE20194 datasets with 130 TNBC patients (46 pathologic complete response, 84 residual disease) were used as the external validation cohort. To identify the significant gene set and develop a predictive model to discriminate pCR and RD groups, we first used Lasso and Elastic-Net Regularised Generalised Linear Models using the R package “glmnet” on the 300 markers to identify the best combination with the greatest predictive power. Then, we used the 10-fold cross-validation method to evaluate the discrimination ability, between pCR and RD, to obtain a relatively unbiased estimate. After the LASSO regression analysis, a predictive model based on 20 was used to fit a generalised linear model. The predictive capability was measured by the receiver operating characteristic curve (ROC curve) area under the curve (AUC) using the R package “pROC”. Results were evaluated using the area under the ROC curve. The optimal model was selected by maximising AUC. The model was tested on data with known and unknown chemotherapy responses using the function predict.glm with the ideal lamda as the s variable.

### TNBC subtyping

The TNBCtype web-based tool (http://cbc.mc.vanderbilt.edu/tnbc/) was used to classify each TNBC patient sample. Subtyping was performed on RNA expression data, normalised within TNBC patients as recommended by the tool, from each patient.

### H3K27ac ChIP-seq analysis

Eight primary TNBC patients as well as corresponding transcriptome (RNA-seq) datasets were downloaded from ENA: accession number PRJEB33558. Reads were aligned to the human genome (GRCh38) using Bowtie2. H3K27ac ChIP peaks were identified by the MACS version 2 software package with paired input samples with the callpeak function using default settings, genome set to ‘hs’, and peak calling set to—broad. Differential enrichment of peaks between pCR and RD was called using the package diffbind with the criteria of a minimum of 50% overlap of peaks.

### Super enhancer identification and analysis

Samples were merged based on their subtyping using bedtools merge and enhancer and SE elements were mapped and quantified by MACS and ROSE software^46^. ROSE analysis was performed with default parameters of 12.5 kb stitching distance, and TSS exclusion size set to 0, consistent with prior studies, we did not exclude TSS elements^75^. Using the output SE bed file from ROSE we identified regions unique to each TNBC subtype using ChIPpeakanno with 50% overlap of SE regions. SE-associated genes were identified by ROSE by assigning the discovered SEs to the nearest genes. Hierarchical clustering on SE-associated uniquely expressed genes was performed using Euclidean distance metric and Ward’s linkage method and plotted using the R package “ComplexHeatmap”. Colour bars for associated pathway data for each subtype were determined using EnrichR.

### Hi-C analysis in TNBC patients

Samples were obtained from GSE167150 and processed using HiCExplorer^76^. Reads were aligned to the human genome (GRCh38) using Bowtie2. Then the HiCExplorer pipeline was implemented with default parameters.

### TNBC chemoresistance CRC reconstruction

We performed the core transcriptional regulatory circuitry analysis using CRC mapper (https://github.com/linlabcode/CRC) as previously described^77^. Within Super Enhancer regions, the CRC software uses FIMO to find enriched (*q* value < 1e−5) TF motif occurrences. CRC first identified TFs that are active, regulated by a proximal, overlapping, or the closest SE region. The total degree is a measure of how often a given TF participates in a regulatory interaction with other TFs. It is defined as the number of unique TFs participating in a regulatory interaction that affects a given TF plus the number of unique TFs that are regulated by a given TF.

### Genetic dependency

Gene expression for each CRC TF was extracted from TNBC cancer cell lines from CCLE in DepMap (https://depmap.org/portal/). To identify genetic dependencies of subtype-specific CRC TFs, Achilles gene effect scores and dependency scores were downloaded for each subtype of TNBC cell lines screened by RNAi and CRISPR from DepMap. We built linear regression models of each TF correlation strength and viability of each subtype across all TNBC cell lines tested. T-statistic testing was used to evaluate association strength between subtype correlation strength and viability.

### Cell culture

The TNBC lines HCC1806 and HCC70 were maintained in RPMI 1640 (Gibco, 21875034) medium supplemented with 10% FBS, 1% glucose and 1 mM sodium pyruvate (Thermo, 11360070). MDA-MB-468, MDA-MB-453, and MDA-MB-231 cells were maintained in DMEM (Dulbecco’s modified Eagle’s medium) with 10% FBS. Cells were grown as monolayers at 37 °C in a humidified CO_2_ (5%) incubator.

### siRNA transfection

The scrambled siRNA control and ON-TARGETplus SMARTpool siRNA targeting human TFAP2C, TFAP2C, SP1, STAT3, TCF7L2, PRDMI, and FOSL2 were purchased from Dharmacon. Transfection was performed using Lipofectamine™ RNAiMAX (Invitrogen, 13778150) according to the manufacturer’s instructions. In brief, cells were seeded at 180k/well for MDA-MB-231, MDA-MB-453, HCC1806 and HCC70. Cells were seeded at 250k/well for MDA-MB-468 cell lines. All cells were seeded the day before the transfection. siRNA at a final concentration of 5 pmol was diluted in 45 μl of Opti-MEM (Gibco, 31985047) and 2.25 μl of Lipofectamine RNAiMAX was diluted in 45 μl of OPTI-MEM. The diluted siRNA and Lipofectamine RNAiMAX were mixed and incubated at room temperature for 10 min. Ninety microliters of transfection mixture were added to each well of 12-well plates. Twenty-four hours later, the transfection cocktail was replaced with complete media for each cell line.

### RNA isolation and RT-qPCR

Total RNA was isolated from cells in culture using Trizol reagent (Ambion, 15596018) according to the manufacturer’s instructions. RNA concentration and purity were measured using the NanoDrop Spectrophotometer. cDNA was synthesised using Verso cDNA synthesis kit (Thermo, 01280858). RT-qPCR was performed in the SybrGreen programme: 5 min pre-incubation at 95 °C; amplification 45 cycles at 95 °C for 10 s, 60 °C for 10 s and 72 °C for 10 s; melting was performed at 95 °C for 5 s, 65 °C for 1 min, 97 °C on hold; final cooling was performed at 40 °C for 30 s. Results were analysed and normalised by the relative quantity (ΔΔCt) method. Wilcoxon Rank Sum and Kruskal–Wallis tests were used to calculate significance.

### MTT assay and drug sensitivity analysis

siRNA-transfected cells were cultured for 24 h and treated with Epirubicin and Docetaxel at the desired concentration for each cell line. DMSO served as vehicle control. The treated cells were incubated for 48 h, and a cytotoxicity assay was performed using an MTT assay kit (Roche, 11465007001) according to the manufacturer’s protocol. Briefly, 10 μl MTT (5 mg/ml) was added to each well and allowed to form formazan crystals for four hours in the incubator. 100 μl of solubilisation solution was added to each well and incubated overnight in the incubator in a humidified atmosphere. The next day, complete solubilisation of the purple formazan crystals was confirmed and then the absorbance values were determined using a microplate reader (BMG FLUOstar Omega) at 590 nm. The experiments were repeated twice, and data are represented as mean ± SD from three technical replicas. Wilcoxon Rank Sum and Kruskal–Wallis tests were used to calculate significance.

### Statistical analysis

All the statistical analyses were performed using R (version 4.1.1) and GraphPad Prism 9. Student’s *t*-test, Wilcoxon rank-sum test and Kaplan–Meier were utilised in this study. *p*-values of <0.05 were considered statistically significant (*p < 0.05; **p < 0.01; ***p < 0.001).

### Reporting summary

Further information on research design is available in the Nature Research Reporting Summary linked to this article.

### Supplementary information

Supplementary figures

Supplementary Data 1

Supplementary Data 2

Supplementary Data 3

REPORTING SUMMARY

## Data Availability

Accession numbers for all publicly available datasets used are in Supplementary Data 1 Sheet 1.

## Code availability

The merged microarray datasets and all scripts used in this study are located at: Github.com.
