# Supplementary material for: Decoding gene regulatory circuitry underlying TNBC chemoresistance reveals biomarkers for therapy response and therapeutic targets
Source: NPJ Precis Oncol. 2024 Mar 12;8:64. doi: 10.1038/s41698-024-00529-6 (PMC10933292; doi:10.1038/s41698-024-00529-6)
Supplement: Supplementary file 1 — Supplementary figures [file 41698_2024_529_MOESM1_ESM.pdf]

Supplementary Figure 1

Lusby et al

a

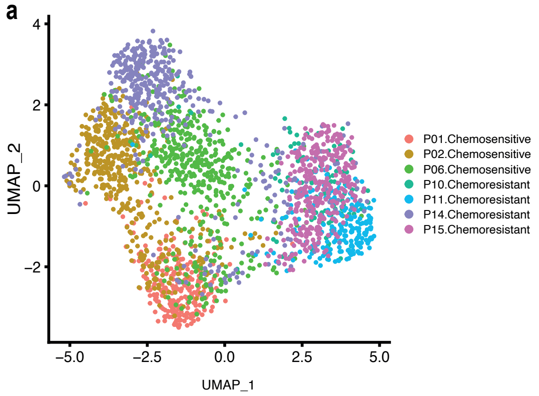

b

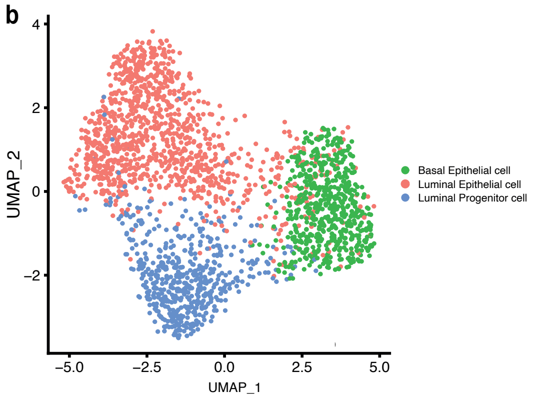

c

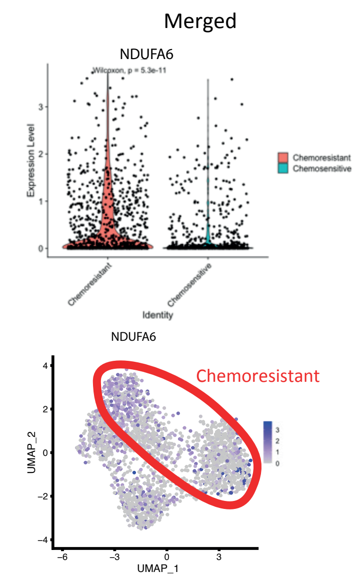

d

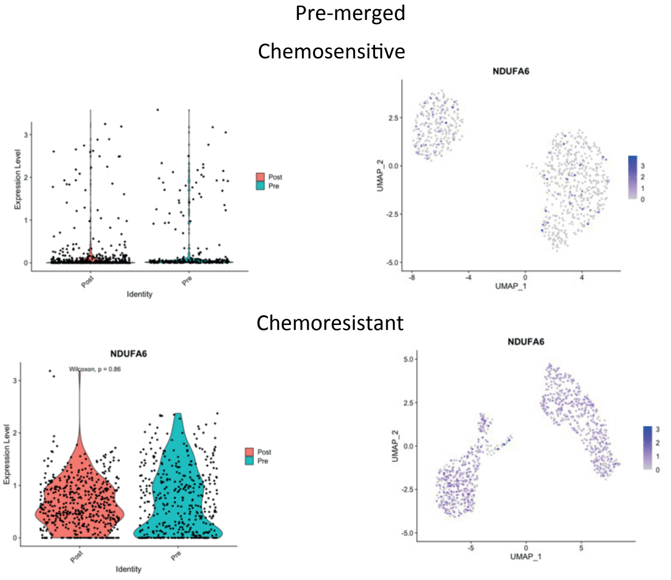

e

Sensitive Pre

Term	P-value
purine nucleobase biosynthetic process (GO:0009113)	9.20E-06
positive regulation of phagocytosis (GO:0050766)	1.08E-05
B cell receptor signaling pathway (GO:0050853)	1.15E-05
neutrophil degranulation (GO:0043312)	1.58E-05
base-excision repair, gap-filling (GO:0006287)	1.58E-05
neutrophil activation involved in immune response (GO:0002283)	2.02E-05
pyroptosis (GO:0070269)	2.05E-05
mitotic sister chromatid segregation (GO:0000070)	2.12E-05
microtubule cytoskeleton organization involved in mitosis (GO:1902850)	2.34E-05
neutrophil mediated immunity (GO:0002446)	2.42E-05

Sensitive Post

Term	P-value
positive regulation of transcription, DNA-templated (GO:0045893)	4.38E-04
cellular response to hormone stimulus (GO:0032870)	4.42E-04
regulation of cell proliferation involved in heart morphogenesis (GO:2000136)	4.64E-04
regulation of dendritic cell apoptotic process (GO:2000668)	4.81E-04
negative regulation of apoptotic signaling pathway (GO:2001234)	5.45E-04
positive regulation of transcription by RNA polymerase II (GO:0045944)	5.79E-04
peptidyl-threonine dephosphorylation (GO:0035970)	0.00102166
fat cell differentiation (GO:0045444)	0.00118821
negative regulation of dendritic cell apoptotic process (GO:2000669)	0.0015363
neuron migration (GO:0001764)	0.0021862

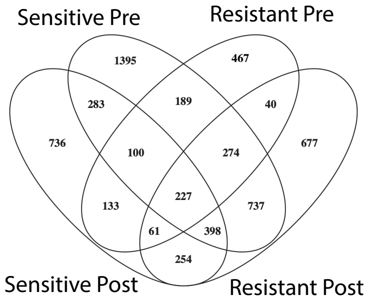

Resistant Pre

Term	P-value
negative regulation of ERBB signaling pathway (GO:1901185)	1.14E-04
blood vessel endothelial cell migration (GO:0043534)	1.52E-04
negative regulation of epidermal growth factor receptor signaling pathway (GO:0042059)	1.80E-04
positive regulation of nervous system development (GO:0051962)	2.01E-04
positive regulation of cell junction assembly (GO:1901890)	6.13E-04
positive regulation of DNA replication (GO:0045740)	0.00103701
regulation of epidermal growth factor receptor signaling pathway (GO:0042058)	0.00128173
axo-dendritic transport (GO:0008088)	0.00210086
positive regulation of transferase activity (GO:0051347)	0.00214639
epidermal growth factor receptor signaling pathway (GO:0007173)	0.00244335

Resistant Post

Term	P-value
protein N-linked glycosylation (GO:0006487)	7.74E-05
wound healing (GO:0042060)	1.13E-04
chordate embryonic development (GO:0043009)	1.29E-04
extracellular structure organization (GO:0043062)	1.38E-04
somatic recombination of immunoglobulin genes involved in immune response (GO:0002204)	1.41E-04
in utero embryonic development (GO:0001701)	1.47E-04
isotype switching (GO:0045190)	3.49E-04
negative regulation of nuclear division (GO:0051784)	3.49E-04
external encapsulating structure organization (GO:0045229)	4.20E-04
glycosaminoglycan biosynthetic process (GO:0006024)	4.21E-04

f

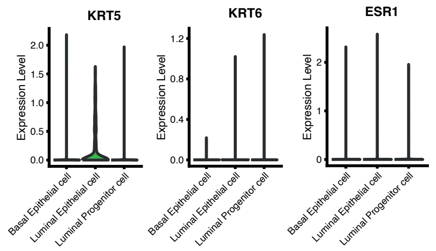

Supplementary Figure 1. Merged Pre-Treatment Samples and Gene List Selection
a) Uniform Manifold Approximation and Projection (UMAP) visualization featuring chemoresponsive and chemosensitive patients, with clear patient labels. b) UMAP visualization with annotations indicating different cell types. c) Expression analysis of NDUFA6 within the merged data, with statistical significance assessed using the Wilcoxon rank-sum test (p=5.3e-ll). d) Expression analysis of NDUFA6 within the unmerged data, evaluated for statistical significance using the Wilcoxon rank-sum test. e) Gene Ontology (GO) terms associated with all markers identified in pre/post chemoresistant and chemosensitive patients. f) Expression of luminal markers across cell types in pre-treatment TNBC chemoresistant and chemosensitive patients.

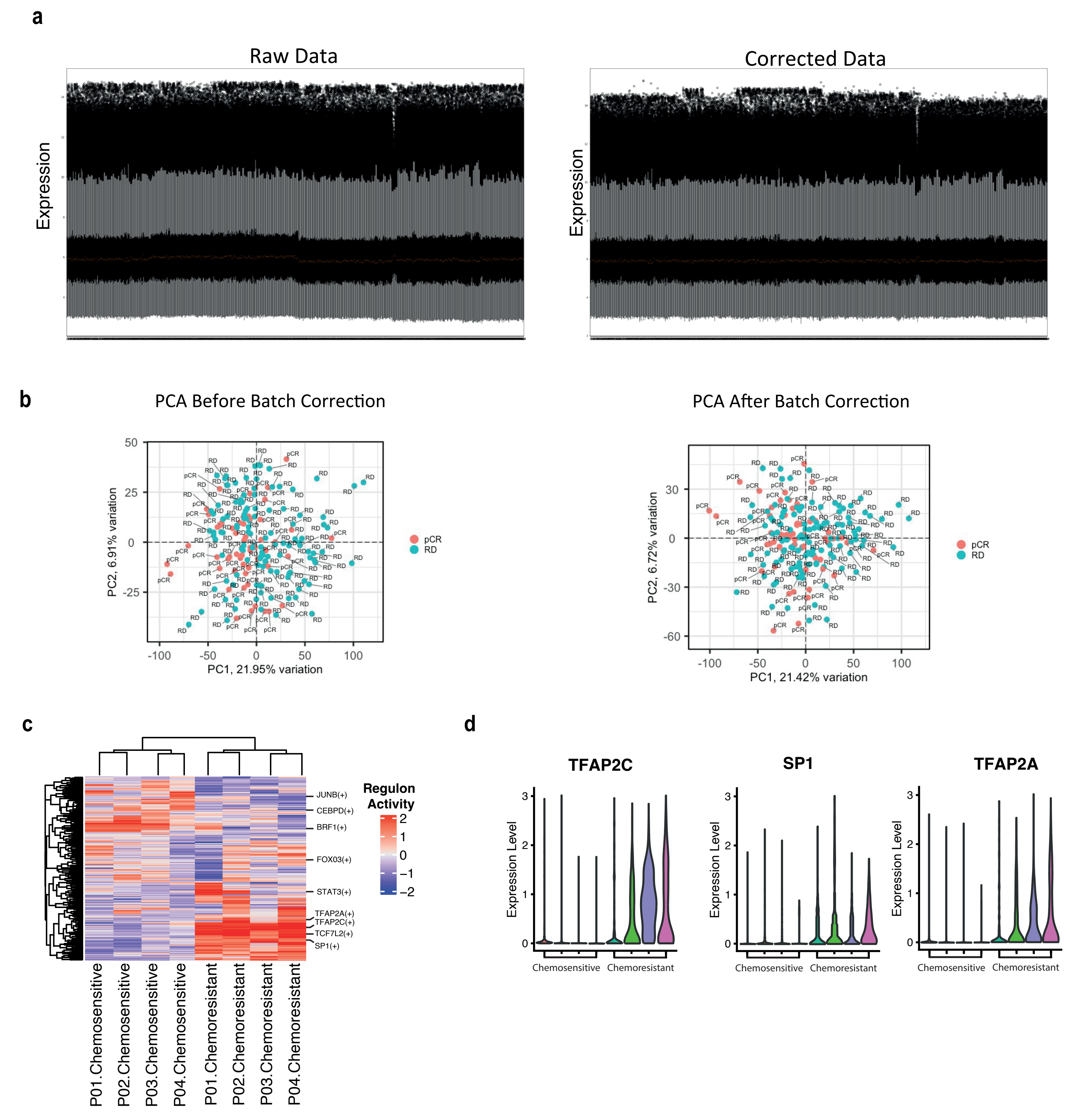

Supplementary Figure 2. Quality Control Figures from microarray batch correction and per-patient expression of chemoresistant TFs a) Comparison of gene expression values before and after batch correction. b) Principal Component Analysis (PCA) plots illustrating the distribution of samples before and after batch correction. c) Heatmaps of Significant Top Regulators: Heatmaps display significant top regulators based on the Area Under the Curve (AUC) score for chemosensitive and chemoresistant patients. The top motifs, based on averaged binary scores are labelled d) Feature Plots showing expression of TFAP2C, TFAP2A and SP1 in TNBC scRNA-seq across patient subtypes.

Supplementary Figure 3

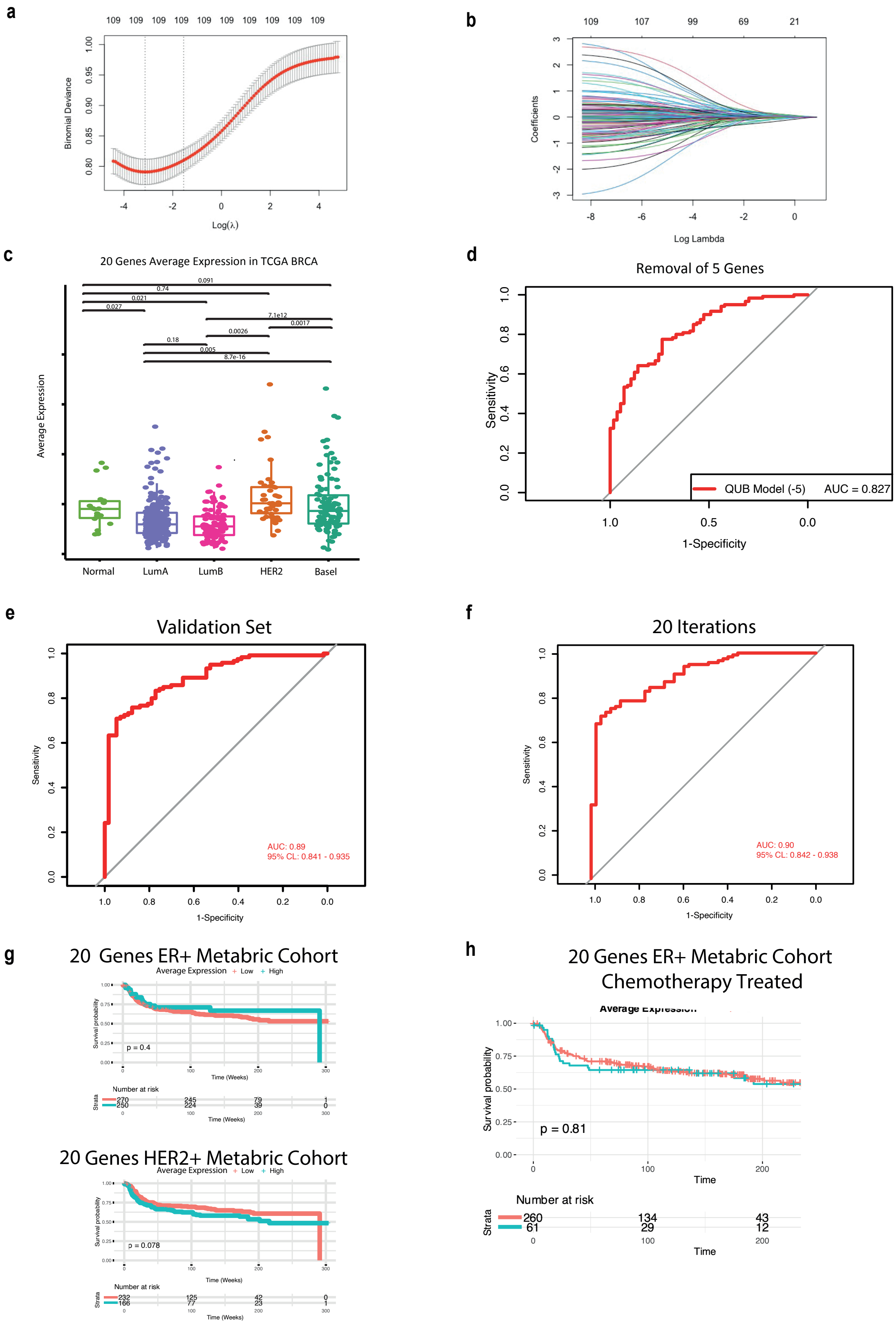

Supplementary Figure 3. Development and Validation of the 20 Gene Model
a) Selection of the tuning parameter (λ) using 10-fold cross-validation with the LASSO model. The vertical lines indicate λ.min and λ.1se, while the red line represents the cross-validation curve plotting mean binomial deviance against log- λ. b) Coefficients of the 20 genes and 21 probe IDs used to construct the predictive model. c) Average expression levels of the 20 genes in the TCGA-BRCA cohort, with statistical significance determined by the Wilcoxon rank-sum test. d) Receiver Operating Characteristic (ROC) curve illustrating model performance in the validation cohort (AUC=0.89). e) ROC curve demonstrating the model's predictive capability after the removal of five genes (AUC=0.678). f) ROC curve displaying the results of 20 iterations of cross-validation, highlighting the model's robustness (AUC=0.90). g) Kaplan–Meier survival plots depicting the survival outcomes of ER+ and HER2+ patients from the METABRIC Cohort based on the 20 gene model. h) Survival Analysis in NAC-Treated ER+ Patients: Kaplan–Meier survival plot revealing the survival outcomes of TNBC patients from the METABRIC Cohort who exclusively received neoadjuvant chemotherapy (NAC) based on the expression of the 20 genes (Log-rank test, p=0.81).

Supplementary Figure 4

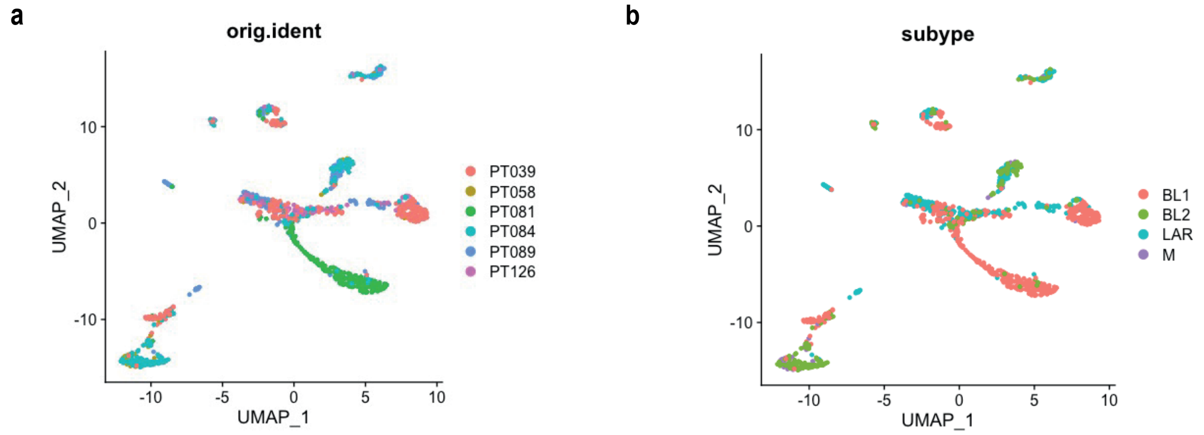

c

Patient	Prediction	Subtype
PT039	RD	BL1
PT081	pCR	M
PT084	pCR	BL1
PT084	RD	BL2
PT089	RD	LAR
PY126	pCR	LAR

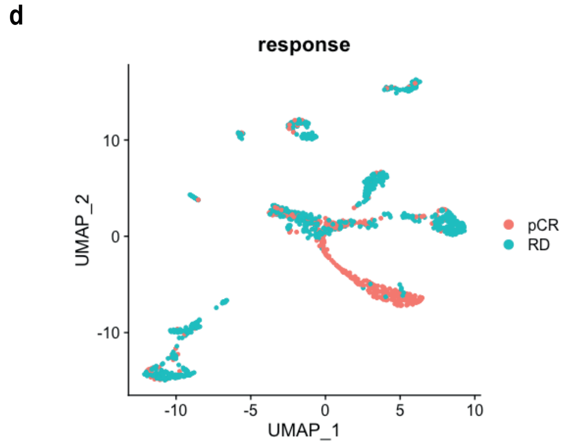

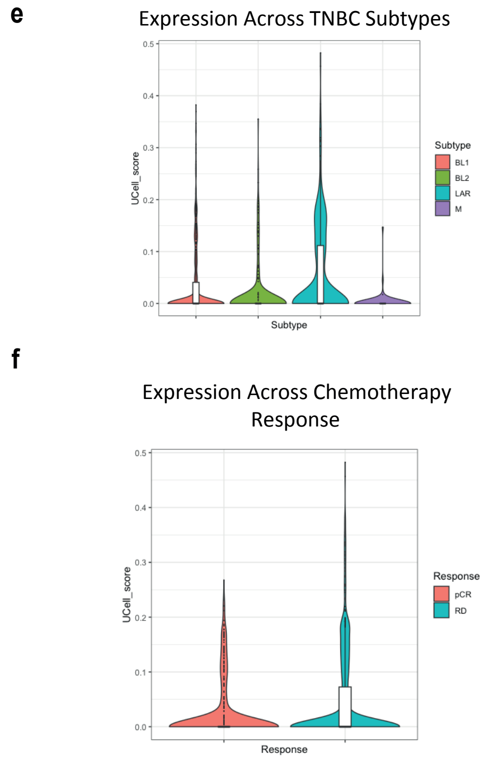

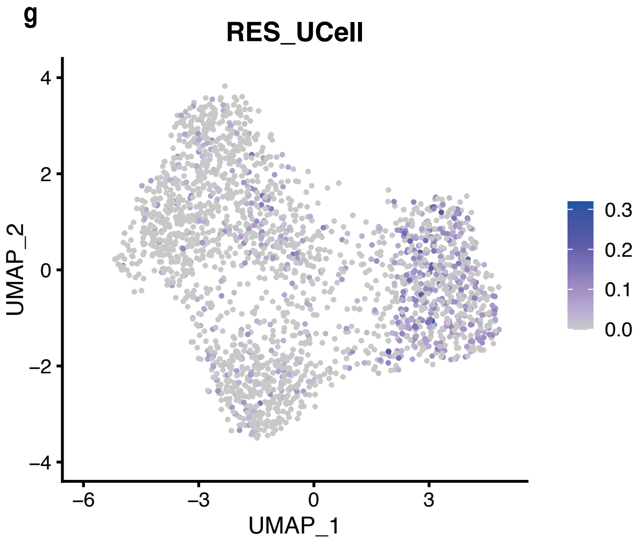

Supplementary Figure 4. Pseudobulk RNA-Seq Analysis of TNBC Patients
a) Clustering of untreated TNBC patients based on gene expression profiles. b) Patient labels according to pseudobulk subtype classification. c) Classification of patients into RD or pCR groups using our gene panel. d) UMAP projection coloured based on our predictive model. e) Expression patterns of the 20-gene panel across different TNBC subtypes f) Expression patterns of the 20-gene panel across different TNBC subtypes. g) UCell scoring of 2 gene signature

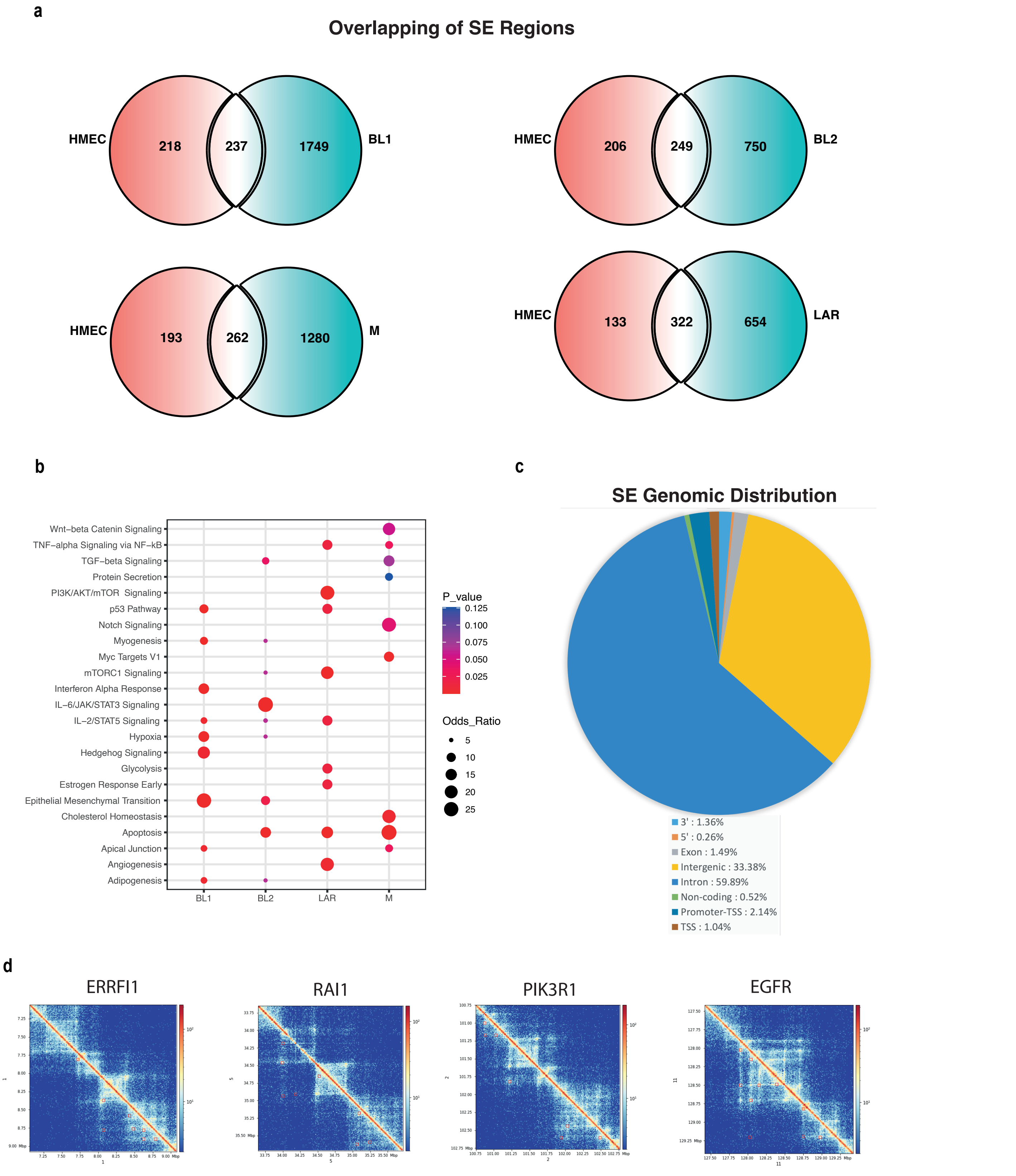

Supplementary Figure 5. Identification of Tumour Specific Super Enhancers
a) Genomic Distribution of Super Enhancers (SEs) in TNBC. b) Venn Diagrams comparing SEs identified in each TNBC subtype with SEs identified in HMEC samples. c) Signalling pathways enriched for subtype-specific SEs. d) Hi-C plots illustrating chromatin loops between SE regions and predicted target genes.

Supplementary Figure 6

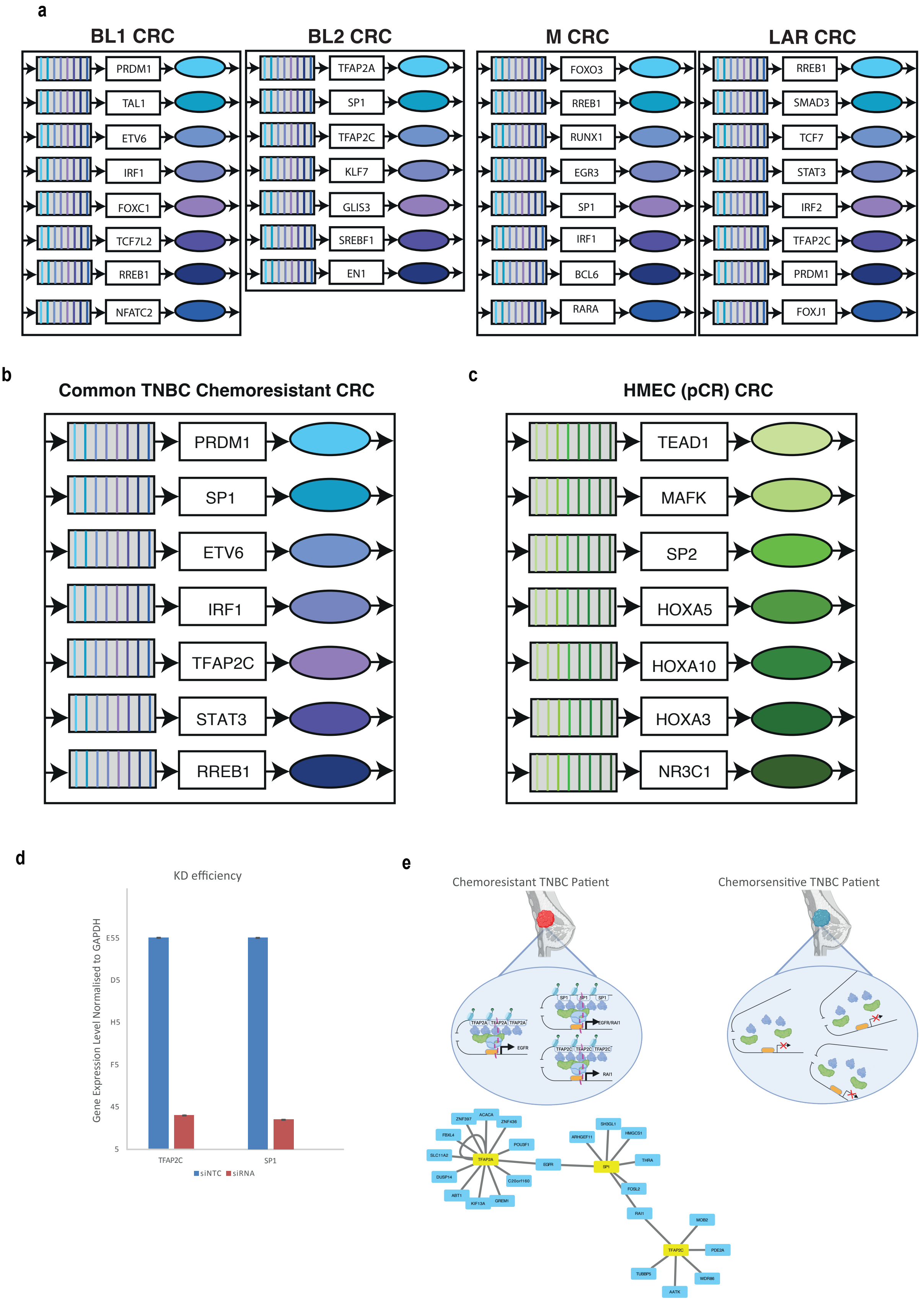

Supplementary Figure 6. CRC Analysis reveals highly connected TFs
a) CRC analysis of all TFs identified for each TNBC patient. b) CRC analysis common across all TNBC subtypes. c) CRC analysis of HMEC samples. d) KD efficiency of TFAP2C and SP1 normalised to GAPDH. e) Schematic illustrating the gene regulatory network (GRN) of transcription factors (TFs) and their target genes identified through SCENIC analysis. The diagram highlights the mechanistic role of super enhancers (SEs) in driving the expression of chemoresistant genes in TNBC chemoresistant patients.
