# Supplementary material for: Decoding gene regulatory circuitry underlying TNBC chemoresistance reveals biomarkers for therapy response and therapeutic targets
Source: NPJ Precis Oncol. 2024 Mar 12;8:64. doi: 10.1038/s41698-024-00529-6 (PMC10933292; doi:10.1038/s41698-024-00529-6)
Supplement: Supplementary file 5 — REPORTING SUMMARY [file 41698_2024_529_MOESM5_ESM.pdf]

nature portfolio

Corresponding author(s): Vijay Tiwari

Last updated by author(s): 20/12/23

Reporting Summary

Nature Portfolio wishes to improve the reproducibility of the work that we publish. This form provides structure for consistency and transparency in reporting. For further information on Nature Portfolio policies, see our Editorial Policies and the Editorial Policy Checklist.

Statistics

For all statistical analyses, confirm that the following items are present in the figure legend, table legend, main text, or Methods section.

n/a	Confirmed	
☐	☒	The exact sample size (*n*) for each experimental group/condition, given as a discrete number and unit of measurement
☐	☒	A statement on whether measurements were taken from distinct samples or whether the same sample was measured repeatedly
☐	☒	The statistical test(s) used AND whether they are one- or two-sided<br>*Only common tests should be described solely by name; describe more complex techniques in the Methods section.*
☐	☒	A description of all covariates tested
☐	☒	A description of any assumptions or corrections, such as tests of normality and adjustment for multiple comparisons
☐	☒	A full description of the statistical parameters including central tendency (e.g. means) or other basic estimates (e.g. regression coefficient) AND variation (e.g. standard deviation) or associated estimates of uncertainty (e.g. confidence intervals)
☐	☒	For null hypothesis testing, the test statistic (e.g. *F*, *t*, *r*) with confidence intervals, effect sizes, degrees of freedom and *P* value noted<br>*Give P values as exact values whenever suitable.*
☒	☐	For Bayesian analysis, information on the choice of priors and Markov chain Monte Carlo settings
☐	☒	For hierarchical and complex designs, identification of the appropriate level for tests and full reporting of outcomes
☒	☐	Estimates of effect sizes (e.g. Cohen's *d*, Pearson's *r*), indicating how they were calculated

Our web collection on statistics for biologists contains articles on many of the points above.

Software and code

Policy information about availability of computer code

Data collection	Only publicly available data was used in this study, All data supporting the findings of this study are available within the article and it's supplementary files provided with this paper
Data analysis	R v4.1.1<br>python v3.10<br>gglot R v3.4.2<br>affy R v1.72.0<br>UCell R v1.3.1<br>SingleCellExperiment R v1.16.0<br>SCENIC R v1.2.4<br>pySCENIC v0.12.0<br>sva R v3.42<br>glmnet R v4.17<br>pROC R v1.18.0<br>TNBCtype web-based tool (http://cbc.mc.vanderbilt.edu/tnbc/)<br>bowtie v1.2.2<br>MACS2 v2.2.9.1<br>Diffbind R v3.4.11<br>ROSE v1.3.1<br>crcMapper https://github.com/younglab/CRCmapper<br>ChIPpeakanno R v3.28.1

ComplexHeatmap R v2.15.1
EnrichR R v3.2
GraphPad Prism 9.4.0
HiCExplorer v3.7.2

For manuscripts utilizing custom algorithms or software that are central to the research but not yet described in published literature, software must be made available to editors and reviewers. We strongly encourage code deposition in a community repository (e.g. GitHub). See the Nature Portfolio guidelines for submitting code & software for further information.

Data

Policy information about availability of data

All manuscripts must include a data availability statement. This statement should provide the following information, where applicable:

- Accession codes, unique identifiers, or web links for publicly available datasets
- A description of any restrictions on data availability
- For clinical datasets or third party data, please ensure that the statement adheres to our policy

All data supporting the findings of this study are available within the article and it's supplementary files provided with this paper

Research involving human participants, their data, or biological material

Policy information about studies with human participants or human data. See also policy information about sex, gender (identity/presentation), and sexual orientation and race, ethnicity and racism.

Reporting on sex and gender	All patients were female breast cancer patients
Reporting on race, ethnicity, or other socially relevant groupings	caucasian
Population characteristics	All patients data contained breast cancer subtype information and treatment outcomes
Recruitment	No patients where recruited fro this study, all data was publicly available
Ethics oversight	Not applicable

Note that full information on the approval of the study protocol must also be provided in the manuscript.

Field-specific reporting

Please select the one below that is the best fit for your research. If you are not sure, read the appropriate sections before making your selection.

☒ Life sciences ☐ Behavioural & social sciences ☐ Ecological, evolutionary & environmental sciences

For a reference copy of the document with all sections, see nature.com/documents/nr-reporting-summary-flat.pdf

Life sciences study design

All studies must disclose on these points even when the disclosure is negative.

Sample size	The analysis was performed on all available TNBC data where patients response to chemotherapy was known.
Data exclusions	No exclusion outside of clinical and gnomic filtering to ensure only TNBC samples where used.
Replication	All data analysis contained a minimum of two replicates
Randomization	Patients where selected based on their response to chemotherapy and placed into two cohorts; pathologic complete response or residual disease. Other covariates where not used to randomize patients as all data was from patients at the pre-treatment stage
Blinding	Blinding was not relevant to this study as patient outcomes where required to facilitate subsequent analysis

Reporting for specific materials, systems and methods

We require information from authors about some types of materials, experimental systems and methods used in many studies. Here, indicate whether each material, system or method listed is relevant to your study. If you are not sure if a list item applies to your research, read the appropriate section before selecting a response.

Materials & experimental systems

n/a	Involved in the study
☒	☐ Antibodies
☐	☒ Eukaryotic cell lines
☒	☐ Palaeontology and archaeology
☒	☐ Animals and other organisms
☒	☐ Clinical data
☒	☐ Dual use research of concern
☒	☐ Plants

Methods

n/a	Involved in the study
☒	☐ ChIP-seq
☒	☐ Flow cytometry
☒	☐ MRI-based neuroimaging

Eukaryotic cell lines

Policy information about cell lines and Sex and Gender in Research

Cell line source(s)	Cal85, HCC70, HCC1806, MDA-MB-453, MDA-MB-231, and MDA-MB-468 were obtained from ATCC
Authentication	None of the cell line used in this study were authenticated
Mycoplasma contamination	Cell lines used not tested for mycoplasma contamination
Commonly misidentified lines (See ICLAC register)	No commonly misidentified cell lines were used
